# Innovations in Air Quality Monitoring: Sensors, IoT and Future Research

**DOI:** 10.3390/s25072070

**Published:** 2025-03-26

**Authors:** Saim Shahid, David J. Brown, Philip Wright, Ahmad M. Khasawneh, Bryn Taylor, Omprakash Kaiwartya

**Affiliations:** 1 Department of Computer Science, Nottingham Trent University, Nottingham NG1 8NS, UK; saim.shahid@ntu.ac.uk (S.S.); david.brown@ntu.ac.uk (D.J.B.); 2 Cobac Security Limited, The Granary, Church Street, Thrumpton, Nottingham NG11 0AX, UK; phil.wright@cobacsecurity.co.uk (P.W.); bryn.taylor@cobacsecurity.co.uk (B.T.); 3 School of Computing, Skyline University College, University City of Sharjah, Sharjah P.O. Box 1797, United Arab Emirates; ahmad.khasawneh@skylineuniversity.ac.ae

**Keywords:** air quality monitoring, sensors, internet of things, air pollutants, sensors design

## Abstract

Recently, Air Quality Monitoring (AQM) has gained significant R&D attention from academia and industries, leading to advanced sensor-enabled IoT solutions. Literature highlights the use of nanomaterials in sensor design, emphasising miniaturisation, enhanced calibration, and low voltage, room-temperature operation. Significant efforts are aimed at improving sensitivity, selectivity, and stability, while addressing challenges like high power consumption and drift. The integration of sensors with IoT technology is driving the development of accurate, scalable, and real-time AQM systems. This paper provides technical insights into recent AQM advancements, focusing on air pollutants, sensor technologies, IoT frameworks, performance evaluation, and future research directions. It presents a detailed analysis of air quality composition and potential air pollutants. Relevant sensors are examined in terms of design, materials and methodologies for pollutant monitoring. A critical review of IoT frameworks for AQM is conducted, highlighting their strengths and weaknesses. As a technical contribution, an experimental performance evaluation of three commercially available AQM systems in the UK is discussed, with a comparative and critical analysis of the results. Lastly, future research directions are also explored with a focus on AQM sensor design and IoT framework development.

## 1. Introduction

Air pollution is a global crisis that adversely affects human health, ecosystems, and the economy. The growing awareness of environmental issues is significantly contributing to people’s desire to better understand the air quality around them [1]. According to the World Health Organisation, outdoor air pollution caused approximately 4.2 million premature deaths in 2019 [2]. Additionally, household air pollution leads to about 3.2 million deaths annually, including over 237,000 deaths of children under 5 years old [3]. Researchers in [4] have estimated that Particulate Matter pollution causes the premature deaths of 48,625 adults annually in the United Kingdom. These alarming figures, coupled with increasing public interest, have generated a demand for Internet of Things (IoT) technology equipped with relevant sensors to provide comprehensive and granular data in order to support public health, regulatory compliance, and environmental protection efforts [5].

**Figure 1:**
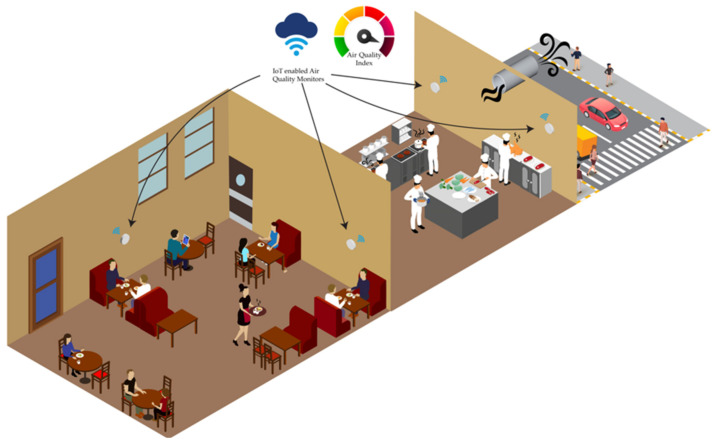
An example of IoT-based AQM system in typical restaurant environment.

Researchers are leveraging IoT and advanced sensors in their efforts to develop accurate, scalable, and real-time AQM systems (see Figure 1) [6]. Investigations are being conducted to design interconnected, battery-efficient solutions to monitor a wide range of pollutants [7]. A key focus of this ongoing research includes miniaturising these devices while maintaining robust calibration, cost-effective manufacturing, and room-temperature operation [8,9]. In the multidisciplinary field of advanced air quality sensor design and fabrication, researchers are working to enhance the sensing materials for pollutant monitoring by improving various sensor parameters [10]. These parameters include power efficiency, operating temperature, humidity dependence, sensor drift, sensitivity and selectivity [11,12]. Additionally, significant efforts are being made to overcome the challenges associated with metal-oxide sensor technology [13]. In the field of IoT, researchers are embedding multiple sensors together to monitor microclimates as well as gather granular multi-point indoor and outdoor pollution data through participatory or crowd-sourced settings [14,15].

The primary objective of these research works is to enhance the emerging technology of AQM. The ultimate goal is to transform the current environment monitoring process into automated, spatiotemporal systems capable of monitoring a broader range of air pollutants in real-time [16]. These advanced systems can significantly improve public awareness of environmental conditions around them in a variety of settings. Indoors, they can be useful in hospitals, factories, social venues, homes as well as in industrial kitchens and more. Outdoors, these devices can be utilised to monitor air quality in parks, traffic zones, rural and urban settings, public transportation, personal vehicles, and even in human space exploration [17].

One of the many challenges faced by researchers is reducing energy consumption in AQM systems [18]. These sensors draw more power as compared to the microcontrollers which significantly reduces the battery life of the overall system [19]. This issue is exacerbated when multiple sensors are used on a microcontroller to measure different pollutants. Many researchers have explored renewable energy sources, such as solar power, to address the power needs of their AQM devices [20]. Additionally, the research community is actively addressing other critical concerns, including accuracy, reliability, and calibration practices, as well as network scalability, security, and cost-effectiveness [21]. Despite significant advancements in AQM, the multidisciplinary design process of intricate sensors remains a distinct field from the integration of these sensors into IoT solutions. Researchers typically focus on either sensor design architecture or IoT frameworks, but not both as a cohesive solution.

In this context, this paper presents a technical insight into the innovations in AQM considering pollutants, sensor designs, IoT frameworks, systems performance evaluation, and future research directions. The following are the key parts and potential contributions of this paper:

1. Technical details of air quality composition and potential air pollutants are presented.
2. AQM sensors are scrutinised in terms of design, materials and methodologies for pollutant monitoring.
3. A critical review of IoT frameworks for AQM is carried out alongside an analysis of their strengths and weaknesses.
4. Experimental performance evaluation of three commercially available AQM systems in the UK is discussed with a comparative and critical results analysis.
5. Future research directions in AQM are also highlighted related to sensor designs and IoT framework development.

The remainder of the paper is structured as follows: Section 2 provides technical information on the composition of the air environment and potential pollutants. Section 3 examines AQM sensors with the emerging materials and methodologies. Section 4 reviews IoT-based advancements in AQM devices, focusing on the integration of multiple pollutant monitoring sensors with various microcontrollers. Section 5 discusses experimental results for evaluating the performance of some commercial AQM devices. Section 6 identifies future research directions in AQM, followed by a conclusion presented in Section 7.

## 2. Air Composition and Pollutants

This section details the typical composition of the air environment and identifies potential pollutants or toxicants along with their key sources. These details are crucial for understanding the AQM research and its significance.

### 2.1. Atmosphere Air Composition

Earth’s atmosphere consists of about 78% Nitrogen and 21% Oxygen, with trace amounts of other gases like Carbon Dioxide (CO_2_), Neon, and Hydrogen, among others. In addition to these gases, the atmosphere holds various particles, including natural aerosols such as dust and pollen, which are carried by the wind. It also transports pollutants like soot and smoke from car exhausts and power plants, with CO_2_ being a significant contributor to human-induced global warming. Moreover, the air is filled with living organisms known as bioaerosols, which are tiny microbial organisms. These organisms cannot fly but can travel long distances through the air, aided by wind, rain, or even a sneeze. To assess air quality, the Air Quality Index (AQI) matrix is used, which assigns values to the cleanliness of the air. Lower AQI values indicate cleaner air. Conversely, when the AQI exceeds 100, often due to factors like forest fires and heavy urban traffic, it can be comparable to inhaling car exhaust throughout the day. Consequently, it is advisable to limit outdoor activities during such conditions [22].

### 2.2. Air Pollutants

#### 2.2.1. Ammonia

Ammonia (NH_3_) is a colourless, pungent gas extensively employed in fertiliser production, cleaning agents, and various industrial processes. It arises naturally through the decomposition of organic nitrogen-containing materials in the environment. Although essential for biological nitrogen cycling, high concentrations can irritate the eyes, skin, and respiratory system. Chronic exposure in occupational settings, particularly agriculture and chemical manufacturing, may lead to long-term respiratory impairment [23].

#### 2.2.2. Particulate Matter

Particulate Matter (PM) consists of tiny inhalable particles, including sulphates, nitrates, NH_3_, salts, black carbon, mineral dust, and water droplets. PM is categorised by its size, with PM2.5 and PM10 being the most significant for health and regulatory purposes. PM10 particles with a diameter of less than 10 µm mainly come from sources like pollen, sea spray, wind-blown dust from erosion, agriculture, roads, mining, construction sites and waste burning. Fine PM2.5 particles with a diameter of less than 2.5 microns originate from fuel combustion in power plants, industries, vehicles, and secondary sources like chemical reactions between gases. Indoor sources of PM include burning fuels in open fires, inadequately ventilated stoves, and space heaters as well as activities like preparing animal feed, heating water, and brewing beverages. The International Agency for Research on Cancer classified PM as a cause of lung cancer in 2013, and it is a key indicator to assess the health impacts of air pollution on a population [24].

#### 2.2.3. Nitrogen Dioxide

Nitrogen Dioxide (NO_2_) is a reddish-brown gas that dissolves in water and acts as a very strong oxidiser. It is mainly produced through the combustion of fuels at high temperatures, which occurs in processes such as heating, transportation, industrial activities, and power generation [25]. Additionally, household sources like furnaces, fireplaces, and gas stoves contribute to NO_2_ emissions. Exposure to this gas can irritate the respiratory tract and worsen existing respiratory conditions. It also plays a significant role in ozone formation at ground level which is a pollutant closely tied to conditions like asthma and respiratory ailments.

#### 2.2.4. Carbon Monoxide

Carbon monoxide (CO) is a colourless, odourless gas produced by the incomplete combustion of carbon-based fuels such as wood, gasoline, coal, natural gas, and kerosene. Common sources include rudimentary cooking devices, open fires, oil lamps, furnaces, fireplaces, and motor vehicles. This gas permeates lung tissues and enters the bloodstream, impairing the body’s ability to bind with oxygen effectively. The resulting oxygen depletion damages tissues and cells, causing respiratory distress, fatigue, dizziness, and flu-like symptoms. Prolonged exposure to elevated CO levels can be fatal [26].

#### 2.2.5. Sulphur Dioxide

Sulphur Dioxide (SO_2_) is a colourless gas with high water solubility and is primarily produced through burning fossil fuels in homes, industrial facilities, and power plants. Exposure to this gas can increase the likelihood of hospital admissions and emergency room visits due to asthma-related complications [27].

#### 2.2.6. Lead

It is found in products like paints, ceramics, pipes, solders, gasoline, batteries, ammunition, and cosmetics, as well as in ambient air from leaded fuel exhaust. Exposure to lead in children can result in behaviour and learning problems, lower IQ, hyperactivity, growth inhibition, hearing impairments, and anaemia, with extreme cases leading to seizures, coma, or fatality [28]. Adults exposed to lead are more susceptible to cardiovascular issues, high blood pressure, hypertension, reduced kidney function, and reproductive issues [29].

#### 2.2.7. Polycyclic Aromatic Hydrocarbons

Polycyclic Aromatic Hydrocarbons (PAH) exist as particulates in the atmosphere and originate from the incomplete combustion of organic materials such as meat cooking, fossil fuels in sources like industrial furnaces, diesel engines, and wood-burning stoves, as well as from tobacco smoke. Short-term exposure to these chemicals can cause irritation in the eyes and respiratory passages. Extended exposure to these substances has been linked to a higher risk of developing lung cancer [30].

#### 2.2.8. Volatile Organic Compounds and Formaldehyde

Volatile Organic Compounds (VOCs) and Formaldehyde (CH_2_O) have high vapour pressure and low water solubility, and are found both indoors and outdoors. They are released as gases from various solids or liquids, often reaching higher indoor concentrations, sometimes ten times more than outdoors. Environmental Protection Agency has revealed that indoor levels of some organic pollutants are two to five times higher than outdoors. VOCs originate from the use and storage of products like cleaning supplies, office equipment, pesticides, paints, adhesives, particleboard, and plywood, etc. [31]. Formaldehyde, a colourless gas with a pungent smell, ranks among the most prevalent VOCs encountered indoors. According to the World Health Organisation, prolonged exposure to this gas has been linked to the development of nasopharyngeal cancer [32].

#### 2.2.9. Ozone

It is a secondary pollutant primarily formed in the lower atmosphere when nitrogen oxides, released from the combustion of fossil fuels, and VOCs react under strong solar radiation. Unlike the protective ozone layer in the stratosphere, ground-level ozone near the Earth’s surface is a major component of photochemical smog [33]. It can cause oxidative damage to the respiratory tract’s lining, leading to eye and nose irritation, reduced lung function, and exacerbations of asthma and Chronic Obstructive Pulmonary disease. Although it is predominantly an outdoor pollutant, ozone can infiltrate indoors, affecting indoor air quality. To protect public health, the World Health Organisation recommends maintaining an 8 h mean ozone concentration below 100 µg/m^3^ which is approximately 50 parts per billion (ppb) [34].

#### 2.2.10. Radon

It is a radioactive gas originating from specific rock and soil formations. This gas tends to accumulate in the lower levels of homes when ventilation and evacuation systems are insufficient. Recent studies conducted in Europe, North America, and Asia have shown that indoor radon exposure is linked to lung cancer. This makes radon the primary contributor to lung cancer in non-smokers across these regions [35].

#### 2.2.11. Hydrogen Sulphide

Hydrogen sulphide (H_2_S) is a colourless, flammable gas recognised for its distinctive rotten-egg odour and is primarily generated by decomposing organic matter. Although trace amounts may play a physiological role, elevated concentrations can be highly toxic by inhibiting cellular respiration. Occupational exposure in industries such as petroleum refining or wastewater treatment can lead to significant health risks, including respiratory and neurological damage. Symptoms at lower concentrations include eye and throat irritation, while higher levels may rapidly induce unconsciousness or death [36].

**Figure 2:**
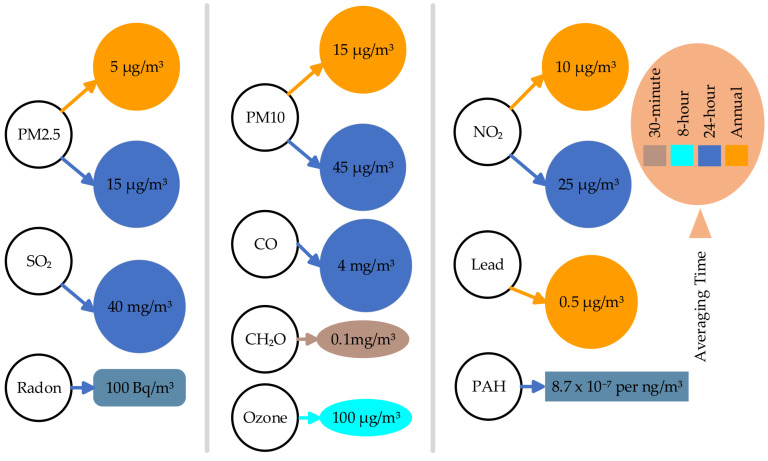
World Health Organisation’s recommended guideline for pollutants.

Figure 2 shows the World Health Organisation’s reference concentrations for various air pollutants based on different averaging periods [37]. Colors are used for time duration. 

The pollutants detailed below do not have specific quantitative guideline limits. However, they are included within the World Health Organisation’s Global AQ guidelines due to their potential health impacts.

#### 2.2.12. Black Carbon

Commonly known as soot, it constitutes a significant portion of PM2.5 and originates from incomplete combustion of fossil fuels, biofuels, and biomass. Emissions come from human activities like diesel vehicles and biomass cookstoves, as well as natural events like wildfires. As a potent greenhouse gas, black carbon contributes to regional ecological disruption and glacier melt. Exposure, both short-term and long-term, is linked to adverse cardiovascular health effects and premature mortality [38].

#### 2.2.13. Ultrafine Particles

These particles, measuring 0.1 micrometres or less in diameter, primarily originate from combustion processes in transportation such as vehicles, aviation, and shipping, as well as in industrial facilities, power plants, and residential heating systems. Exposure to these fine particles heightens the risk of pulmonary, cardiovascular, and ischaemic heart diseases [39].

#### 2.2.14. Mould

It is a type of microscopic fungus that grows in damp places. Moisture accumulation and the subsequent growth of mould and bacteria can result from structural deficiencies in buildings, inadequate heating and insulation, or insufficient ventilation. These conditions lead to the production of allergens and irritants capable of triggering asthma attacks in individuals allergic to mould. Additionally, they can cause irritation in the eyes, skin, nose, throat, and lungs of both mould-allergic and non-allergic individuals [40,41].

**Figure 3:**
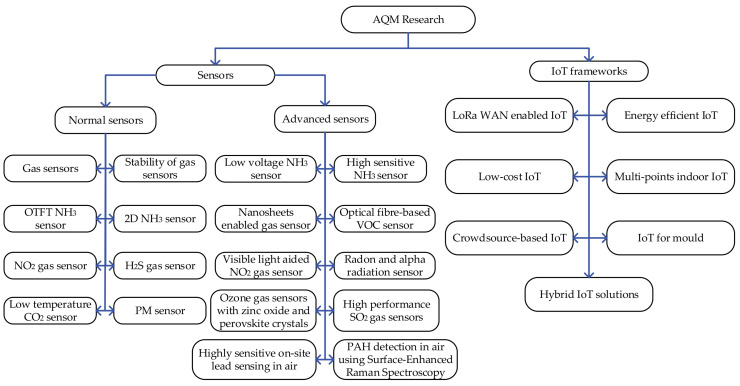
Overview and distribution of topics covered in Section 3 and Section 4.

Figure 3 shows the taxonomy of Section 3 (left-part) and Section 4 (right-part) in our paper, illustrating the distribution and type of papers reviewed.

Sensors are crucial components in AQM devices, as they detect and quantify critical air parameters. Therefore, in the next section, we examine the design and construction of AQM sensors. It focuses on established materials such as metal-oxide semiconductors and emerging materials like nanomaterials, alongside advanced manufacturing techniques, including Organic Thin Film Transistor (OTFT) technology. Desirable properties such as room-temperature operation are highlighted, as they enhance practicality and reduce energy consumption. The design and parametric evaluation of key pollutant sensors such as NH_3_, H_2_S, VOC, NO_2_, CO_2_, Radon, SO_2_, Ozone, PM, Lead, and PAH will further illustrate how these sensors can be integrated into modern IoT frameworks for AQM, thereby bridging the gap between sensor development and real-world IoT architecture for AQM.

## 3. Sensors for AQM

### 3.1. Gas Sensors

The significance of graphene oxide and other two-dimensional (2D) materials for gas-sensing applications has been discussed [42]. Notably, 2D materials have a high surface-to-volume ratio because these materials are only a few atoms thick which makes them promising for gas sensor applications. Molybdenum disulfide, Tungsten disulfide, Tungsten diselenide, and Molybdenum diselenide exhibit a bandgap that can be tuned by changing the number of layers, which is useful in the construction of nanomaterials and gas-sensing devices. However, these materials require high temperatures to function thus affecting device longevity, and their drawback lies in the lack of nuanced selectivity, compromising their ability to precisely distinguish between different gases.

When CO undergoes oxidation on a p-type sensing material, it loses an electron to fill a hole. Since holes are the majority charge carriers in p-type material, the introduction of an electron to fill a hole leads to electron-hole recombination. This, in turn, reduces the number of positive charge carriers (which are the majority charge carriers in a p-type material), thereby decreasing the conductivity of the gas-sensing material and increasing electrical resistance. The authors discuss how chemiresistors alter their electrical resistance when relevant gas molecules adsorb onto the sensing layer. They explore field effect transistor-based gas sensors and their semiconductor channel response to the target gas, impacting the drain-source current. Additionally, they mention impedance sensors, highlighting these sensors’ ability to enhance the selectivity of the target gas by utilising a sinusoidal waveform of a specific frequency. Observing the resultant waveform’s behaviour allows for drawing conclusions. Monitoring parameters such as frequency, capacitance, and resistance improve the determination of the sensed gas, enabling impedance gas-sensing devices to detect sub-parts per million (ppm) concentrations. The authors also discuss optical gas sensors, highlighting their capability to alter optical properties like absorbance, fluorescence, and reflectivity to indicate the presence of a target gas. Subsequently, a detector captures the reflected or diffracted light to convert it into an electrical signal. Furthermore, they delve into Quartz Crystal Microbalance gas sensors, noting that any change in mass on the surface of the quartz crystal due to the adsorption of target gas molecules affects the resonant frequency of the quartz crystal, which can then be measured to identify the presence of the gas.

### 3.2. Stability of Gas Sensors

**Figure 4:**
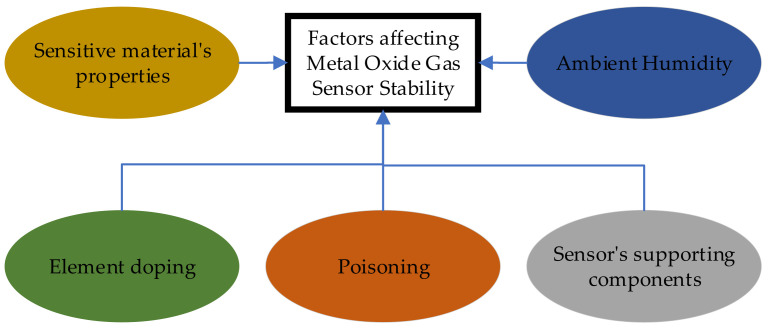
Factors affecting the stability of Metal-Oxide gas sensors.

The stability of gas sensors fabricated using metal-oxide semiconductors has been investigated [43]. Sensor stability is defined as the capability to maintain a consistent and repeatable signal over an adequate duration. The progress in research pertaining to the stability of metal-oxide sensors spanning the period from 2016 to 2021 was comprehensively examined. Metal-oxide semiconductors are widely used due to their attractive characteristics like small size, easy operation, low cost, etc. The sensitivity and selectivity of these sensors are the primary areas of research. Metal-oxide surfaces also usually remain stable when interacting with oxygen in air at high temperatures. Several key factors affecting the stability of semiconductor metal-oxide sensors have been identified (see Figure 4). These include the properties of the sensitive material, element doping, poisoning, ambient humidity, and the supporting components of the sensor, such as integrated heaters used to maintain the semiconductor gas-sensing metal-oxide material within the temperature range of 150 to 450 degrees Celsius.

### 3.3. 2D NH_3_ Sensor

The development of a novel composite made from polymers and 2D materials is detailed [44]. A poly(3-hexylthiophene)/molybdenum disulphide (P3HT/MoS_2_) nanocomposite is synthesised by utilising a bottom gate top contact organic field effect transistor configuration. The active sensing layer was produced using the floating film transfer method (FTM). The study found that within a closed chamber under ambient conditions, this organic field effect transistor with nanofiber morphology showcased a mobility of 0.147 cm^2^/V^−s^ and a threshold voltage of −3.78 volts in air. Upon exposure to 100 ppm of NH_3_ gas, this threshold voltage shifted to −10.71 volts. The device exhibited a limit of detection of 904 ppb. The sensor exhibited a significant sensing response of 63.45% when exposed to 100 ppm concentration of NH_3_ gas. No permanent changes occurred in the sensing layer during the test. Their organic field effect transistor device demonstrated a strong linear response to various concentrations of NH_3_ gas, indicated by a coefficient R^2^ = 0.9964. The response properties of the sensor to methane, CO, and CO_2_ at 100 ppm confirmed that their sensor is highly selective to NH_3_ gas.

### 3.4. Low-Voltage NH_3_ Sensor

A Low-Voltage OTFT-based toxic NH_3_ gas sensor operating at room temperature is fabricated and designed using a self-aligned technique where the gate is made of a highly doped silicon substrate [45]. In this technique, the gate serves as a mask for the subsequent doping of the source and drain regions through the ion implantation method. This method results in more precise channel lengths, improved transistor performance and reduced overlap capacitance between the gate and the source/drain regions. In contrast, gate metals like aluminium or gold in the non-self-aligned techniques cannot withstand the annealing temperatures (above 800 °C) needed for the ion implantation process to restore the crystalline structure of the material.

The capacitance per unit area of the dielectric material is enhanced to achieve 486 nF/cm^2^ at 1 kHz. The newly developed material, LaZrO_x_, demonstrated a low leakage current density of about 0.5 × 10^−8^ A/cm^2^ at −2 V, indicating high capacitance and excellent insulation properties, making it promising for low voltage OTFT applications. The sensor responded to NH_3_ gas at a rate of 47% for concentrations of 5 ppm, with a detection threshold of approximately 11.65 ppb. The sensor typically takes around 9 s to respond and approximately 50 s to recover. Its performance was stable under varying humidity conditions, particularly between 30% and 70% relative humidity. The authors used the following equation to calculate the drain current I_DS_ in the saturation region of their produced NH_3_ sensor:

(1) $$I_{Ds}={µ}_{p}C_{d}\frac{W}{2L}{\left(V_{GS}-V_{th}\right)}^{2};\;\;\;V_{DS}\geq V_{GS}-V_{TH}$$

${µ}_{p}$ represents the mobility, $C_{d}$ denotes the capacitance per unit area of the gate oxide film, W/2L is a width-to-length ratio of the channel, $V_{GS}$ refers to the Gate-Source voltage, $V_{TH}$ is the minimum Gate-Source voltage required to create a conductive channel between the drain and source terminals, and $V_{DS}$ is the Drain-Source Voltage.

### 3.5. Highly Sensitive NH_3_ Sensor

A highly sensitive NH_3_ sensor is developed that is capable of operating at room temperature [46]. Bi-layer metal-oxide dielectric i.e., Titanium dioxide/Hafnium dioxide, is used to enhance the capacitance to $0.926\;\mu F/{cm}^{2}$, so that more charge can be stored at a given gate voltage. This enabled the sensor to turn on at a lower applied gate voltage of −1.5 volts. The layers of Titanium dioxide and Hafnium dioxide are grown sequentially on a boron-based, highly p-doped silicon substrate (gate) which improved the dielectric constant to 42 as compared to 3.9 of SiO_2_. Using the fully solution-processed FTM method, the authors grew gold (Au) doped P3HT film on top of the dielectric layer to act as an organic semiconductor channel. The designed sensor demonstrated a response time of 5 s and a recovery time of 17 s. Additionally, it exhibited a 55% sensing response at a concentration of 5 ppm of the NH_3_ analyte. The following equation is used to calculate the sensing response of their fabricated OTFT-based sensor:

(2) $$S\%=\frac{\left|I_{DS(AIR)}-I_{DS({NH}_{3})}\right|}{I_{DS(AIR)}}\times 100\%$$

where, $I_{DS(AIR)}$ is the drain-source current in normal air and $I_{DS({NH}_{3})}$ the drain-source current when the sensor is exposed to the NH_3_ analyte.

### 3.6. OTFT NH_3_ Sensor

**Figure 5:**
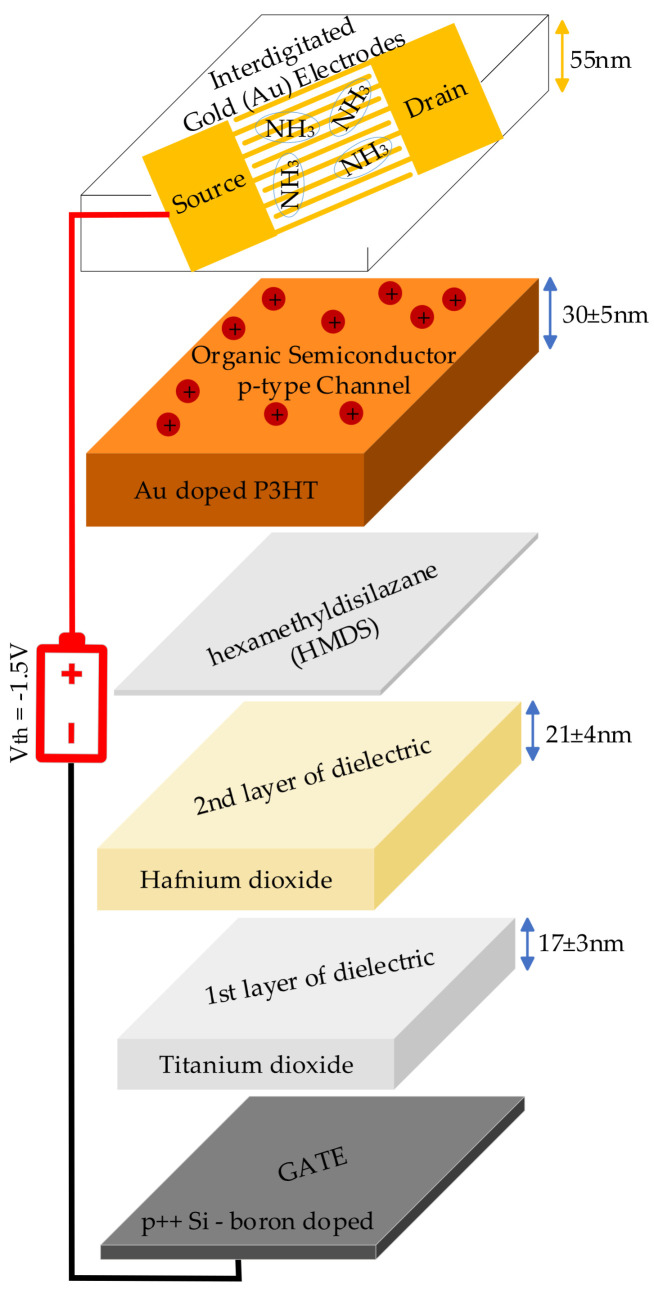
Structure illustration of the OTFT-based low-voltage NH_3_ sensor.

A flexible, low voltage and solution-cast OTFT for NH_3_ sensing at room temperature is developed by utilising an inorganic oxide/polymer-based dielectric layer and polymer/2D nanocomposite-based active gas-sensing layer [47]. As shown in Figure 5, the dielectric layer consists of ZrO_x_/poly (methyl methacrylate)/poly melamine co-formaldehyde, while the active layer comprises P3HT/graphitic carbon nitride. To overcome the inherent brittleness of pristine inorganic oxide dielectrics, the authors merged them with polymers, forming an inorganic oxide-polymer nanocomposite. This novel composite serves as a dielectric material, exhibiting a high dielectric constant of 20. Their sensor demonstrated a low detection limit of 0.5 ppm and a 69% sensing response at 20 ppm of NH_3_. The researchers were able to achieve a low gate threshold voltage of −0.1052 volts in their sensor design.

**Figure 6:**
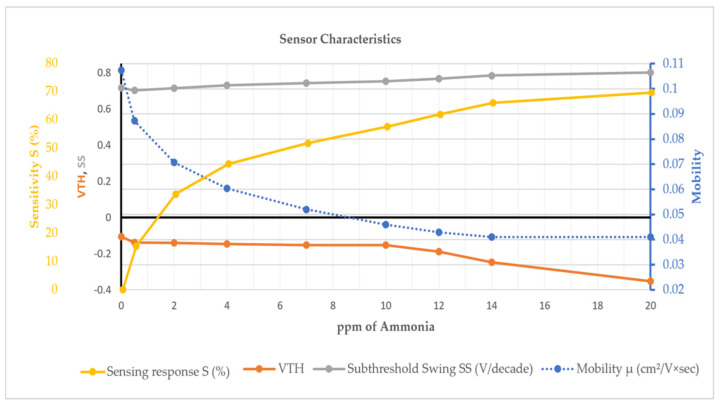
Variation of sensor parameters with respect to NH_3_ concentration.

Figure 6 presents a detailed analysis of the sensor characteristics developed by the authors for detecting NH_3_ concentrations in ppm. This graph illustrates how four key parameters, sensitivity response, threshold voltage, subthreshold swing, and mobility, change with varying NH_3_ concentrations. Sensitivity response (S%), shown in yellow colour, increases as the NH_3_ concentration rises, indicating that the sensor’s responsiveness to NH_3_ improves at higher concentrations. Threshold voltage (V_TH_), depicted in orange colour, shows a decreasing trend with increasing NH_3_ concentration. Subthreshold swing (SS), represented in grey colour, measures the sensor’s switching efficiency in the subthreshold region and shows a rising trend with increasing NH_3_ concentration. Mobility (μ), depicted in blue colour with a dotted line, shows a decrease as NH_3_ concentration increases.

### 3.7. Nanosheets Enabled Gas Sensor

The sensing of NH_3_ gas in p-channel thin-film transistors is enhanced by using a nanocomposite composed of P3HT and graphene oxide [48]. The study involved fabricating the device on a boron-doped silicon substrate through FTM. This research included a detailed electrical characterisation of the P3HT/graphene oxide nanocomposite organic field effect transistor, which was compared with a similar organic field effect transistor that used only P3HT. The nanocomposite organic field effect transistor with palladium electrodes demonstrated an increased response of about 63% at 80 ppm NH_3_ gas. Improvements in crystallinity and grain size in the active layer led to enhanced response and recovery times of approximately 44 s and 82 s, respectively. The characterisation of the film revealed that the P3HT/graphene oxide nanocomposite possessed a higher root mean square roughness compared to pristine P3HT, which, according to the authors, is beneficial for the sensor’s performance due to an increased surface-to-volume ratio. As the trap charge density varies with the changing concentration of NH_3_, the authors have utilised the following equation to calculate the trap carrier density $\left(\Delta e_{trap}\right)$ of the active semiconductor sensing layer.

(3) $$\Delta e_{trap}=\frac{Q_{trap}}{q}=\frac{\Delta V_{TH}C_{ox}}{q}$$

q is elementary charge constant which is $1.602\times 10^{-19}Coulombs$. $Q_{trap}$ represents the trapped charge. $\Delta V_{TH}$ represents the threshold voltage shift, which is indicative of voltage change at the gate required to maintain a constant current, depicting the concentration of NH_3_. $C_{ox}$ is the capacitance per unit area of the gate oxide.

### 3.8. H_2_S Gas Sensor

A novel sensor is designed for detecting H_2_S gas at room temperature [49]. The sensor utilises a nanocomposite of P3HT and Graphene Quantum Dot in its active sensing layer, which is applied using FTM on a SiO_2_-coated p++ Si substrate. The sensor demonstrated a 91% sensing response and an 18% shift in gate threshold voltage at a concentration of 25 ppm of H_2_S, with a very slow recovery time of 225 s. The sensing response at V_GS_ = V_DS_ = −40 volts is calculated using the following equation:

(4) $$S\left(\%\right)=\left|\frac{I_{DS,air}-I_{DS,H_{2}S}}{I_{DS,air}}\right|\times 100$$

where $I_{DS,air}$ is the drain-source current in the normal air conditions, and $I_{DS,H_{2}S}$ is the drain-source current when a concentration of H_2_S is present in the air. The addition of nanomaterial in the conducting polymer has enhanced the current driving capability of the H_2_S sensor by improving the charge carrier transport mechanism. The thickness of the active layer as 30±4 nm, and the quantum dots are approximately 2 nm in size. The surface roughness of the P3HT/Graphene Quantum Dot sensing film is higher compared to the pristine P3HT-based film, which increases the surface-to-volume ratio, thereby enhancing the interaction sites for H_2_S gas. Over a two-week period, the sensor exhibited a response variation of 4% at 54% relative humidity. Additionally, a change in sensing response of 4% to 5% was observed when the relative humidity was increased from 20% to 70%.

### 3.9. Optical Fibre-Based VOC Sensor

**Figure 7:**
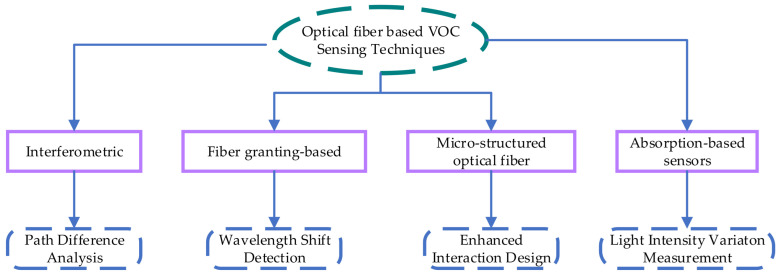
Different methods to sense VOCs using optical fibre technology.

Optical fibre-based sensors for VOC detection exhibit heightened sensitivity, enabling effective detection at sub-ppm levels. Having high vapour pressure, VOCs can evaporate easily into the atmosphere at room temperature. In [50], multiple recent VOC sensor technologies are discussed, including Electrochemical Gas Sensors, Quartz Crystal Microbalances, metal-oxide semiconductors, Non-Dispersive Infrared Gas Sensors, and Colorimetric Gas Sensors. The study notes that metal-oxide semiconductor-based sensors operate at high temperatures and are highly sensitive to external humidity, while Electrochemical Gas Sensors are prone to zero drift and ageing. Optical fibre-based sensors are superior to traditional methods as these sensors are tiny, robust, responsive, and unaffected by drift. The optical fibres typically comprise silica cladding and a germanium-doped silica core. Key issues such as the need for enhanced selectivity, and the development of more compact, and cost-efficient sensors are also highlighted. Future research could benefit from integrating advanced materials and nano-engineering techniques to further improve the sensors’ performance. Figure 7 illustrates various techniques for measuring VOCs and the underlying principles they employ.

### 3.10. NO_2_ Gas Sensor

The traditional methods for sensing NO_2_, which is acidic in nature and has a pungent smell, face several challenges [51]. These challenges include the requirement for high temperatures, prolonged recovery periods, and a decline in performance under harsh conditions. This research explores the application of MoS_2_ for NO_2_ gas sensors, highlighting its ease of integration with different materials, compatibility with a range of devices, adjustable morphology, and ample surface area to facilitate the adsorption of NO_2_ molecules. Several methods for sensing low concentrations of NO_2_ include electrochemical sensors, optical sensors, and resistive sensors [52,53].

The metal-oxide semiconductors, known for their cost-effectiveness and high sensitivity in diverse nanostructures, substantially improve the functionality of NO_2_ gas sensors. However, these semiconductors face challenges in selectivity. It is highlighted that reduced graphene oxide is an outstanding material for constructing NO_2_ sensors, owing to its large surface area and minimal noise characteristics. The authors discussed the significant benefits of using conducting polymers for detecting highly toxic NO_2_ gas, including their ease of manufacture and room-temperature operation, while also acknowledging drawbacks such as degradation and high humidity dependence.

Various techniques are explored for enhancing MoS_2_-based NO_2_ gas sensors, including the use of heterostructures and nanocomposites. Mechanically exfoliated MoS_2_ modified with lead sulphide is investigated, noting that it performs better than sensors using intrinsic MoS_2_ [54]. This research also explains the properties of lead sulphide and MoS_2_. In p-type materials such as lead sulphide, the fermi level is closer to the valence band, indicating a higher concentration of holes in the valence band. Conversely, in n-type materials like MoS_2_, the fermi level lies closer to the conduction band, suggesting a higher concentration of free electrons in the conduction band. When a heterojunction forms between n-type MoS_2_ and p-type lead sulphide, the difference in Fermi levels drives electrons from the n-type MoS_2_ (with a higher Fermi level) to the p-type lead sulphide (with a lower Fermi level). As electrons move from MoS_2_ to lead sulphide, they leave behind positively charged holes in MoS_2_. This migration of electrons continues until an equilibrium is established. At equilibrium, the Fermi levels of MoS_2_ and lead sulphide align, resulting in the formation of a built-in electric field at the junction. This electric field acts to balance the movement of charge carriers, preventing any net flow of electrons or holes across the junction at equilibrium.

### 3.11. Visible Light-Aided NO_2_ Sensor

A NO_2_ detector utilising zinc oxide activated by Cadmium Sulphide is developed [55]. The sensor was fabricated through a simple, rapid, and low-cost Liquid Plasma Spray technique. Cadmium sulphide served as an activator for zinc oxide at room temperature with the aid of visible light. The creation of the cadmium sulphide-zinc oxide sensor involved using an aqueous mixture of cadmium sulphide and zinc acetate as precursors. The authors utilised the narrow-band-gap semiconductor cadmium sulphide to reduce the band gap of zinc oxide, which is at 3.37 electron volts. This is because photogenerated electron-hole pairs in the semiconductor are only produced if the energy of the photon from the projected light is greater than the material’s band gap energy. The band gap of zinc oxide prior to the addition of cadmium sulphide indicates that the material requires ultraviolet light for activation. Therefore, the authors added cadmium sulphide to decrease the band gap, thus enabling the sensor’s activation in visible light. This will help the semiconductor to sense the gases at low temperatures. The authors used lights with wavelengths of 480 nm (blue colour), 510 nm (green colour), and 640 nm (red colour) to study the NO_2_ sensor’s behaviour.

For evaluating the sensor, the researchers utilised a custom setup in which they injected 10 ppm of NO_2_ and synthetic air at flow rates of 25 mL/min and 225 mL/min, respectively, into the testing chamber to achieve a concentration of 1 ppm of NO_2_. The authors employed the surface depletion model and double Schottky barrier model to explain the gas-sensing mechanism. In the proposed model, zinc oxide functions as an electron acceptor, while cadmium sulphide acts as an electron donor. When exposed to light, particularly blue or green wavelengths, cadmium sulphide absorbs high-energy photons. This absorption process leads to the formation of photogenerated electron-hole pairs. As a result, the electrical resistance of the material reduces as compared to when it is in a darker environment.

### 3.12. Low-Temperature CO_2_ Sensor

**Figure 8:**
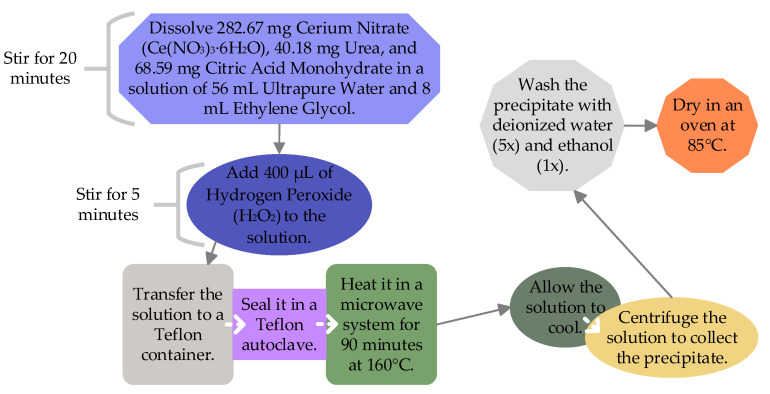
Step-by-step process for developing yolk-shell CeO_2_ nanosphere powder.

The metal-oxide-based chemo-resistive CO_2_ sensors are widely used and show potential but there is a need for a CO_2_ sensor that is both efficient and capable of operating at low temperatures [56]. An efficient resistive CO_2_ sensor has been developed using hollow nanostructured cerium dioxide (CeO_2_), a type of metal oxide. The sensor operates at a low temperature of 100 °C and was tested under relative humidity conditions ranging from 30% to 70%. The yolk-shell nanospheres they developed exhibit twice the sensitivity, improved stability and reversibility compared to ceria nanoparticles available in the market. Additionally, these sensors demonstrate quicker response times and a higher CO_2_ adsorption capacity. The authors describe a simple approach to create a hollow and porous structure of the yolk-shell nanoparticles for improved gas diffusion and larger specific surface area, resulting in superior electrical and sensing properties. Figure 8 shows the step-by-step process used by the authors to develop yolk-shell CeO_2_ nanosphere powder.

The authors developed two types of sensors for comparison. One sensor incorporated their custom-made yolk-shell hollow CeO_2_ powdered nanospheres, while the other used commercially available CeO_2_ nanoparticles. To fabricate these CO_2_ sensors, they mixed CeO_2_ with ethylene glycol to create a paste. This paste was then applied to an alumina substrate equipped with interdigitated Pt electrodes on the top and a Pt heater located on the bottom to heat the sensor to 100 °C. Following this, the sensors were annealed at 400 °C for an hour to eliminate any residual ethylene glycol and stabilise the sensing layer. The authors used the following formula to calculate the sensing response of their developed sensor.

(5) $$Sensing\;response=\frac{R_{{CO}_{2}}}{R_{0}}$$

where $R_{{co}_{2}}$ is the resistance of the sensor at a specific concentration of CO_2_, while R_0_ is the baseline resistance measured in synthetic air without the presence of CO_2_. The authors assessed the adsorption capacity of their developed sensor material by thermogravimetric analysis. This technique helped in the comparison of the sensing material’s weight before and after interaction with CO_2_. They found that, although the sensor exhibited a good sensing response, its reversibility was compromised when operated at room temperature. Their findings also showed that rising relative humidity decreases the electrical resistance of the sensor, while the baseline resistance remains stable. They concluded that due to their hollow and porous structure, the yolk-shell CeO_2_ nanospheres exhibit a surface area 16 times larger than commercially available CeO_2_ powder. This facilitates efficient diffusion of CO_2_ and the carrier gas into the sensing film, thus also improving recovery time [57].

### 3.13. Radon and Alpha Radiation Sensor

A battery-operated, low-power, cost-effective semiconductor-based sensor is developed to measure radioactive radon (Rn-222) gas and alpha radiation levels in the air [58]. They used a 0.18 μm Complementary Metal-Oxide Semiconductor (CMOS) process to fabricate the sensor. Additionally, they incorporated a layer of polysilicon which is a material composed of many silicon crystals, in contrast to a single continuous crystal structure found in monocrystalline materials. This sensor employs a grid-like structure composed of thousands of parallel floating gate transistors to expand its sensing area. The sensor array consists of 90 × 64 sub-pixels, each measuring 17.5 μm×12.5 μm, resulting in an entire array sensing area of 1.12 mm×1.12 mm. These floating gate transistors, similar to those used in non-volatile memory, retain charge even when the power is off. The floating gates help in trapping and measuring the charge generated by radon radiation. According to the authors, the sensor operates continuously without being affected by humidity.

A Shallow Trench Isolation dielectric layer is used as a sensing capacitor in the sensor where alpha particle interaction will generate electron-hole pairs. The authors calculated that a 0.35 µm thick shallow trench isolation layer absorbs 53.7 kilo electron volts of energy from a 5.5 mega electron volts alpha particle crossing the SiO_2_ layer. Assuming a constant electron-hole pair generation energy of 17 electron volts for SiO_2_, they calculated the number of electron-hole pairs generated $N_{e-h}$ using the following equation.

(6) $$N_{e-h}=\frac{E_{absorbed}}{E_{pair\;generation}}=\frac{53.7\;KeV}{17\;eV}\approx 3150$$

A differential approach is employed by utilising a blind sensor to filter out noise and DC shifts. Multiple layers of Kapton film (50 μm) are applied to shield a portion of the overall sensor, thereby preventing alpha radiation, a heavy particle with limited penetration capability, from interacting with the covered area. This results in obtaining two measurements, one from the normal region of the sensor and the other from the blind region. Both measurements are then compared to eliminate noise.

### 3.14. High-Performance SO_2_ Gas Sensors

A high-performance SO_2_ gas sensor capable of detecting ppm level concentrations has been developed [59]. It utilises a hydrothermal method to synthesise a composite of tin selenide functionalised by graphite-phase carbon nitride. At 200 °C, the sensor showed a 28.9% response to 20 ppm of SO_2_ gas. The developed sensor demonstrated high selectivity when tested against interfering gases of Liquefied Petroleum Gas, CO, methane, H_2_S and hydrogen. Similarly, to design a SO_2_ gas sensor for ppm level detection, experiments have also been conducted using various room-temperature ionic liquids, electrode materials and geometries [60].

### 3.15. Ozone Gas Sensors with Zinc Oxide and Perovskite Crystals

Zinc oxide thin films of 300 nm were deposited on silicon and alumina substrates to create a low-power ozone gas sensor operating at room temperature with zero bias [61]. The sensor demonstrated successful detection of ozone concentrations ranging from 55 to 1150 ppb, with a response time of less than 2 s and a recovery time within 15 s. Notably, the authors reported partial film degradation following prolonged exposure to high ozone concentrations of 4500 ppb. Despite this degradation, their sensor remained reliable for long-term indoor monitoring. Likewise, room-temperature ozone sensors based on inorganic mixed-halide perovskite microcrystals with and without manganese doping have been designed and studied [62]. By tuning the bromine-to-chlorine ratio, they showed that bromine-rich compositions show a p-type response to ozone, while chlorine-rich ones exhibit n-type behaviour. Manganese doping further enhanced sensor performance by creating more active adsorption sites, which improves detection sensitivity down to a few parts per billion.

### 3.16. PM Sensor

A novel interdigitated capacitive sensor for real-time monitoring of sub-micron and nanoscale PM has been introduced to overcome the limitations of conventional optical and gravimetric methods [63]. The sensor utilised capacitance shift analysis to enhance detection sensitivity. It consisted of a disposable chip (2 mm × 12 mm) integrated into an air sampling cassette and a reusable readout board for real-time capacitance measurement. A microheater was incorporated to stabilise readings against environmental variations such as humidity and airflow. Experimental validation showed that 77% of the collected particles were sub-micron, confirming the sensor’s capability for fine PM detection. The authors demonstrated that the sensor response is proportional to particle volume, and a comparison with gravimetric methods revealed that only 1/1000 of the total collected particle mass contributed to the sensor response due to radial deposition effects.

### 3.17. Highly Sensitive On-Site Lead Sensing in Air

A photoluminescent sensor based on a perovskite semiconductor has been developed to detect the presence of lead in the air [64]. In this method, lead ions interact with methyl ammonium bromide, resulting in the formation of a highly luminescent perovskite material. The authors wrote that their sensor can visually detect lead concentrations as low as one nanogram per square millimetre with the naked eye, while digital imaging techniques can identify levels as low as fifty picograms per square millimetre. Similarly, a wearable electrochemical sensor for detecting atmospheric lead has been designed and demonstrated [65]. The sensor was printed on a flexible and transparent vinyl-based substrate to allow direct attachment to the surfaces. On this substrate, they printed three electrodes: a carbon working electrode, a carbon counter electrode, and a silver/silver chloride reference electrode. The working electrode was modified with a bismuth film and a thin Nafion layer to enhance sensitivity and selectivity toward Pb. Lead detection was performed using square wave anodic stripping voltammetry with a portable, miniaturised potentiostat. They reported that their sensor detected lead at levels as low as 50 micrograms per litre.

### 3.18. PAH Detection in Air Using Surface-Enhanced Raman Spectroscopy

A method based on surface-enhanced Raman spectroscopy for detecting PAHs in air has been developed [66]. The Raman signal was enhanced by modifying glass fibre filters with silver nanoparticles to improve the sensitivity and selectivity of the detection process. This study focused on detecting PAHs, specifically fluoranthene, phenanthrene, and pyrene, by capturing them from the air using the coated glass fibre filter. A laser was used to analyse the collected pollutants. The method achieved low detection limits, ranging from 9.11 to 18.18 ppb. The analysis was fast and completed within one minute. The method showed high accuracy and repeatability, with recovery rates ranging from 83 to 126 percent. It was reported that this method is more portable alternative to traditional techniques such as gas chromatography-mass spectrometry. It allows for real-time monitoring of air pollution. The technique is cost-effective, and suitable for field applications.

Comparative analysis of sensor design parameters from various studies is presented in Table 1.

In the next section, we have critically reviewed recent literature on IoT frameworks for AQM. It discusses a range of IoT device designs, up-to-date research findings in terms of issues and solutions, monitoring parameters, and associated sensor-based IoT network architectures.

## 4. IoT Frameworks for AQM

### 4.1. LoRa WAN Enabled IoT

LoRa WAN-enabled IoT framework has been presented in the Mobile Citizen Measurements and Modelling: Air Quality and Urban Heat Islands project [67]. By employing cost-effective sensors, this research aims to narrow the divide between regional and individual measurements. They introduced a distinctive design based on mobile sensing nodes capable of monitoring temperature, humidity, and various pollutants including NO_2_, PM1, PM2.5, and PM10. The authors then conducted a thorough evaluation of their design’s energy consumption and determined that the results met their expectations satisfactorily. The authors utilised LoRa WAN for long-range communication. They have designed the nodes to be mobile to enable participatory monitoring. In their study, sixteen mobile sensor nodes were built to monitor the air quality. Each node also included an integrated fan for the PM monitor which, according to their observations, consumes 77 mA. Additionally, a user-friendly web interface was developed to display the air quality data.

Likewise, an IoT edge device, RnProbe, is designed for integrated radon risk management [68]. This system leverages LoRa WAN and Wi-Fi communication technologies to continuously monitor radon levels alongside other indoor air quality parameters such as temperature, humidity, atmospheric pressure, and CO_2_. It performs edge computing to initially process sensor data locally, ensuring efficient data transmission to a cloud-based analytics platform. The device operates in different modes to optimise power consumption. It draws approximately 160 mA in active mode with all components powered, about 101 mA in sleep mode with partial component shutdown, and up to 330 mA when acting as a gateway due to increased communication demands. The authors reported that their device demonstrates the potential for scalable deployment and real-time monitoring to support radon mitigation strategies and improve indoor air quality.

### 4.2. Low-Cost IoT

Low-cost IoT solutions have been discussed detailing their functional and architectural mechanisms [69]. They highlighted that with proper calibration in specific field conditions, these sensors can achieve coefficient R^2^ values up to 0.99. However, without such calibration, these values may drop to as low as 0.5. The authors discussed methods to improve PM observation quality by integrating regulatory station data with traffic and satellite information. However, they noted that these methods are insufficient for accurately modelling PM levels in urban areas. They highlighted recent advancements where researchers have developed networks of inexpensive, in-field-operated PM sensors. These sensors, working alongside regulatory stations, self-calibrate themselves by using data from these stations throughout their operational lifespan.

**Figure 9:**
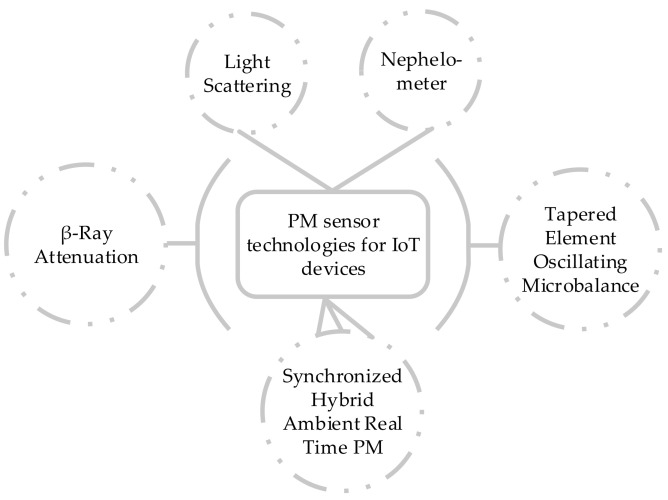
PM measurement techniques.

A smart, low-cost IoT-based system for real-time monitoring of hazardous air pollutants, including hydrocarbons, lead, nickel, and PM has been proposed [70]. Their system used cloud storage, and machine learning-driven predictive analysis to help industries and regulatory bodies track pollution levels, generate alerts, and take preventive measures to reduce environmental and health risks. Researchers in [71] reported that the observed level of PM in bars and pubs where smoking is permitted was 287 µg/m^3^, compared to 34 µg/m^3^ in places where smoking is prohibited. In [72], the authors noted that during cooking, PM generated mainly consists of fine or ultrafine particles and the median mass diameter of these particles does not go above one micron. Deodorisers commonly used in homes are also harmful to the respiratory system, as they produce particles smaller than 2 microns [73]. Figure 9 shows the multiple technologies discussed to measure PM values.

Similarly, an IoT-based smart shirt equipped with an affordable electrochemical sensor has been proposed for continuously monitoring H_2_S levels in hazardous environments [74]. The wearable device wirelessly transmits real-time H_2_S concentration data to a cloud platform using Bluetooth Low Energy via a smartphone gateway. The authors emphasised the sensor’s compactness and notably low-power consumption of 1.86 mW. Additionally, their smart shirt includes an energy harvesting system combining solar, thermal, and piezoelectric sources, generating up to 216 mW. This enables complete energy autonomy and extended operational lifetime which significantly enhances energy efficiency and practicality.

### 4.3. Crowdsource-Based IoT

A crowdsource-Based IoT framework has been investigated for Monitoring Fine-Grained Air quality [75]. The issue of inadequate AQM infrastructure in urban environments has been addressed, highlighting how the scarcity of monitoring nodes severely limits the resolution of air quality data at a city-wide scale. To monitor the dynamic air quality of the city, they employed a fleet of 500 vehicles across Beijing utilising DiDi corporation network to monitor the dynamic air quality of the city. This study was based on the rationale that the air quality inside a vehicle closely resembles that of the external environment through which it travels. The authors developed an algorithm to monitor air quality as vehicle windows are opened and the environment stabilises, i.e., when the concentration gradient between the inside and outside air diminishes. IoT technology was used to transmit the data to the cloud. The developed algorithm initially determines the status of the window and air conditioner by monitoring PM2.5 and humidity levels inside the vehicle. Once the pollutant levels stabilise after opening the windows, the algorithm begins to store and transmit the data.

To smoothen the data, authors have utilised double and triple exponential smoothing equations for PM2.5 and humidity, respectively. This method filters out noise and extracts trends or patterns from a time series. Each data point is represented as x_t_, starting from time t. The following equations were used to model their study, where ∝ is the smoothing factor, which can vary from 0 to 1, and s_(t − 1)_ denotes the previous smoothed value.

(7) s0=x0 st=∝xt+1−∝st−1,     t>0.

The results obtained by the authors show an acceptable bias of 3.64–4.2% from the expected values.

### 4.4. Energy Efficient IoT

It is highlighted that the current AQM systems are precise and responsive, but they come with significant energy operating costs and require expensive, time-consuming laboratory analyses [76]. To address these challenges, the authors introduced a cost-effective metal-oxide-based AQM system for assessing the levels of CO, NO_2_, and SO_2_ in urban environments. They also employed laser diffraction technology to measure PM levels and equipped the device with weather monitoring sensors. In the design, the authors utilised several key components which include a solar-powered rechargeable battery, a graphic user interface, a server for storing information, a microcontroller, and a GPRS module. The device calculates the air pollutant standard index and incorporates an algorithm based on RSSI to reduce packet losses by 9.8% to 11.6% in medium to poor network conditions. To calculate AQI, the authors have used the following equation.

(8) $$I_{p}=\frac{I_{Hi}-I_{Lo}}{{BP}_{Hi}-{BP}_{Lo}}\left(C_{p}-{BP}_{Lo}\right)+I_{Lo}$$

**Figure 10:**
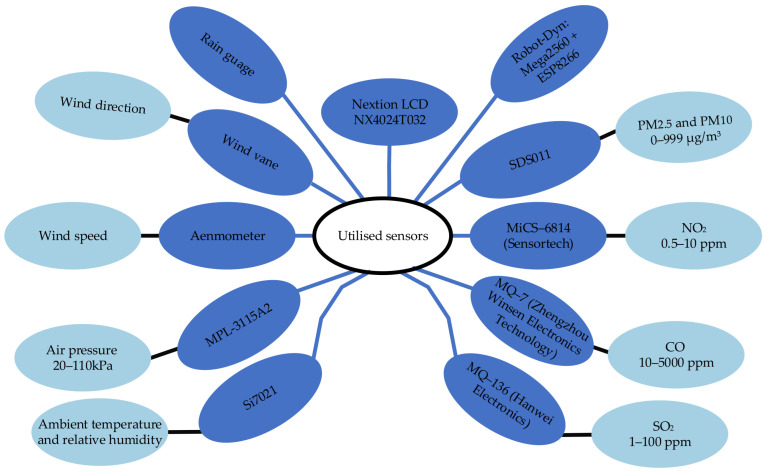
Types and models of Sensors utilised for AQM.

Here, I_p_ represents the AQI corresponding to the concentration of a specific pollutant C_p_. BP_Hi_ and BP_Lo_ are the upper and lower concentration breakpoints, respectively, for the pollutant range. I_Hi_ is the AQI at the higher concentration breakpoint and I_Lo_ is the AQI at the lower concentration breakpoint. They measured the current consumption of the device to be varying between 280 and 410 mA which is significantly higher than that of the transmission module. The authors found PM sensors and the fan are the primary contributors to this increased energy usage. Figure 10 illustrates the integration of various sensors and microcontrollers used in the system. The diagram displays the types of sensors, their specific models, and the environmental parameters they measure. It shows Nextion LCD employed for user interface, and a Robot-Dyn Mega2560 with ESP8266 for processing and connectivity.

Similarly, an energy-efficient IoT-based NH_3_ monitoring system for flexible and wearable applications has been designed in [77]. The authors developed a 3.3 V battery-powered, Wi-Fi-enabled platform operating at room temperature with a low-power consumption of around 262.5 mW, enabling real-time data transmission and cloud-based analytics. Its flexible electronics allowed easy integration into personal wearables, facilitating continuous health and environmental monitoring.

### 4.5. Multi-Points Indoor IoT

Recognising that people spend around 90% of their time indoors, an IoT-based Indoor Air Quality Detector has been developed to monitor key indicators such as CO_2_ levels, PM2.5, temperature, and humidity [78]. They installed seven of these devices throughout a building for a one-month study conducted during the winter season to gather and process data. These devices were connected using Zigbee wireless modules. A central gateway was utilised for data acquisition via Modbus Remote Terminal Unit protocol every two minutes from the sensor nodes which then uploaded the information to the cloud via GPRS/4G connectivity. The users accessed this data via a web or mobile application. The study found that PM2.5 levels surged, increasing by up to ten times during cooking sessions. Additionally, CO_2_ levels were observed to rise to 2500 ppm, significantly higher than the typical indoor range of 400–1000 ppm. Specifications of Sensors used in [78] are presented in Table 2.

STM32F103C8T6 is chosen as the primary microcontroller unit for their development. This microcontroller typically consumes an average of 25 mA, with an additional low-power mode available to enhance energy efficiency. The DRF1609H Zigbee wireless module was utilised for this experiment due to its low-power consumption (25 mA on average, 18 mA in standby, 20 mA while receiving, and 200 mA during transmission), large-scale network capacity, dynamic routing, and convenient debugging capabilities. The equation below demonstrates how the authors calculated relative humidity from the 16-bit readings taken by the humidity sensor. Here, S_RH_ represents the sensor’s raw humidity value.

(9) $$RH=100\times \frac{S_{RH}}{2^{16}-1}$$

**Figure 11:**
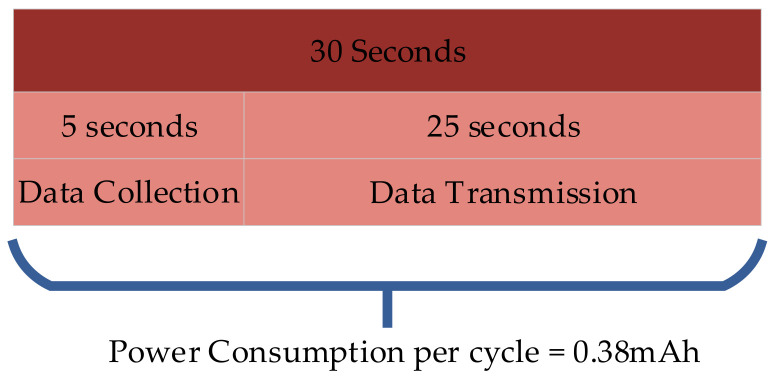
Data collection and transmission intervals with power consumption per cycle.

Figure 11 illustrates the intervals for data collection and transmission, along with the overall power consumption per cycle. The central gateway sends three consecutive acquisition requests to the individual sensor nodes. If the targeted node fails to respond, a packet loss is registered.

This multi-point system was installed across six rooms in a house. The impact of opening and closing windows, as well as human behaviour, was also monitored. Outdoor environment parameters were also recorded for comparison during this experimentation.

This study found that the kitchen had the highest concentration of PM2.5, reaching 1091 µg/m^3^. PM2.5 levels exceeding 250 µg/m^3^ pose a severe health risk and cooking smoke in the kitchen significantly contributes to lung cancer risk. The research revealed that PM2.5 concentrations in the kitchen were 37% higher than in other rooms and increased tenfold during cooking. While CO_2_ levels spiked when there was activity in the kitchen, they also gradually increased in the bedrooms when people were sleeping. Although levels above 1500 ppm are harmful to health, the authors discovered that CO_2_ levels in bedrooms occupied by two adults rose to 2566 ppm, while the room of a 7-year-old reached 1638 ppm, with all doors and windows closed at night.

### 4.6. IoT for Mould

**Figure 12:**
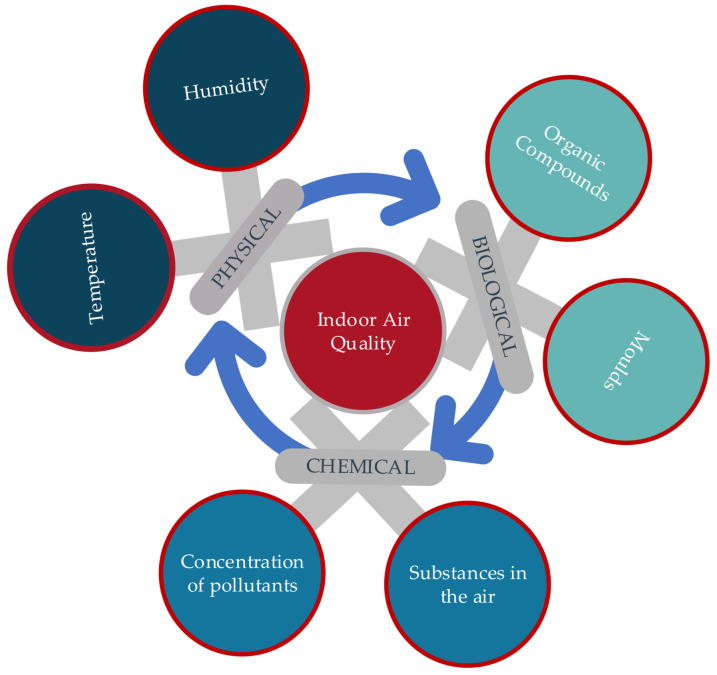
Factors affecting indoor air quality.

IoT framework for mould growth detection has been presented measuring PM2.5, PM10, CO_2_, and Total VOCs [79]. They note that poor design, construction, and maintenance practices of buildings increase the likelihood of mould growth. Additionally, the lifestyle of occupants plays an important role in this process. The resulting mould growth leads to the biodegradation of building materials and significant health issues for the occupants. The study demonstrated that high levels of fungal spores are linked to increased PM2.5, PM10, and CO_2_ levels, resulting in poor indoor air quality. Figure 12 illustrates the physical, biological and chemical factors on which the indoor air quality depends [79].

A standard house with two occupants for two months during the winter period is monitored by employing a range of sensors to monitor air quality. For PM2.5 and PM10, sensors based on the light scattering principle are used. To measure CO_2_ levels, they utilised a non-dispersive infrared sensor. Additionally, they employed metal-oxide semiconductor-based Total VOCs monitoring sensors. Following the monitoring phase, they collected multiple surface and air samples from the house to support their conclusions. Analysis of these samples identified 24 distinct fungal strains. Notably, half of these species were identified as highly harmful to human health, 21% were toxic, and the remainder were associated with long-term health risks. The authors noted that indoor mould affects a much larger area than is visibly apparent. They further stated that while antibacterial products are effective against bacteria, they do not eliminate mould, as mould has a different structure and resilience compared to bacteria. The authors concluded that the peak levels of PM2.5 and PM10 reached $250\;mg/m^{3}$ and $330\;mg/m^{3}$, respectively, significantly exceeding typical annual and 24-h average concentrations. Total VOC levels, however, were not influenced by the presence of mould and remained within normal limits.

### 4.7. Hybrid IoT Solutions

**Figure 13:**
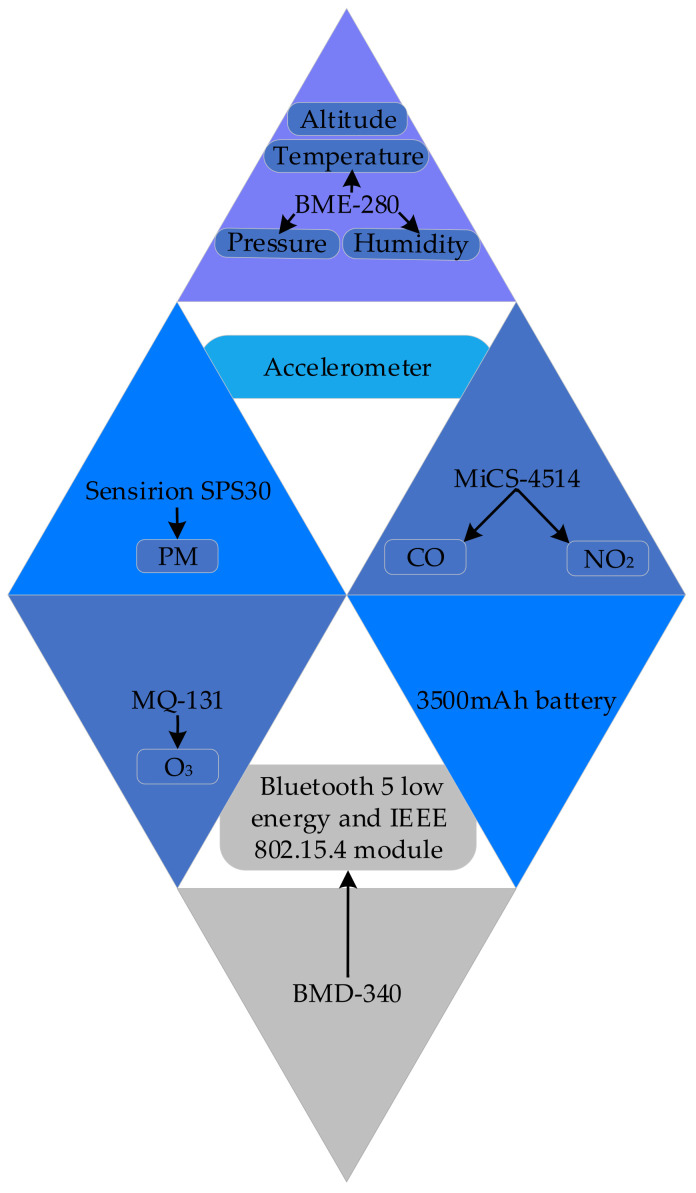
Model numbers and functions of the hardware components.

It is highlighted that despite the costliness of high-end static AQM stations, they remain insufficient in capturing the nuanced fluctuations in an individual’s exposure to pollution [80]. To address this, they have designed a hybrid environment monitoring system capable of operating both indoors and outdoors. Users can wear this sensor on their backpacks, purses, or jackets to understand the air quality around them. The authors have developed an algorithm for their device to differentiate between indoor and outdoor environments. They conducted a comprehensive comparative analysis of their device’s data output against that of established high-end static air quality monitors installed outdoors by the government. By leveraging the power of the crowd, the authors gathered data over a period of two and a half months in the city of Helsinki to analyse the variability of meteorological variables and PM compounds. This system offers personalised data based on an individual’s exposure to everyday pollution. Collected data include PM2.5, PM10, CO, and NO_2_ levels, as well as temperature, relative humidity, light intensity, GPS coordinates, and timestamps. Figure 13 depicts the various sensors and components integrated into the system, along with their respective functions [80].

The authors calculated that their designed device can operate for 22 h on battery power. They justified segregating indoor and outdoor air quality measurements by highlighting significant variations in pollutant types and concentrations between the two environments. They mentioned that not segregating the data will yield oversimplified and inconclusive results. Their experiments revealed that inadequate ventilation resulted in much higher ozone levels indoors compared to outdoors. This phenomenon is likely attributed to different types of indoor air purifiers and electronic devices generating ozone as a by-product. Moreover, they mentioned the continuous spatial variation in PM’s size and chemical composition. They wrote that the PM can consist of nitrates, sulphates, elemental and organic carbon, as well as organic compounds such as PAH, biological entities like endotoxin [81] or cell fragments, and different metals including iron, copper, nickel and vanadium [82].

PM deposition in the lungs of a person depends upon the activity which they are performing in the polluted environment. A person who is driving will experience a lower deposition of PM in their lungs compared to someone exercising in the same environment. The study in [83] also found out that high levels of PM decrease Heart Rate Variability which negatively affects cardiovascular health. The authors wrote that PM deposition in the lungs depends on the living conditions of a person. Their experiments indicate that women are more at risk of PM deposition in their lungs compared to men. Additionally, their device offers real-time air pollution maps to provide insights about the microclimate of a city. The authors concluded that their device can empower people to make informed decisions. They can choose less polluted routes for commutes and plan trips to shopping malls or parks based on the real-time air quality. Table 3 shows comparative assessment and evaluation of various IoT-based AQM Studies.

### 4.8. IoT Devices for AQM-Feature Comparison

The following commercial devices have been used for efficiency testing in real-world conditions in this study. These devices were selected for their availability, suitability for indoor environments and connectivity features.

- Ubibots AQS1 Smart AQ monitor (Arundel, UK) [84].
- Temptop 1000S+ AQ Monitor (London, UK) [85].
- Amazon AQ monitor (London, UK) [86].

Table 4 presents a comprehensive feature analysis of the Ubibots AQS1 Smart AQ monitor, Temptop 1000S+ AQ Monitor, and the Amazon AQ monitor.

These devices enable continuous indoor monitoring without the cost and complexity of regulatory-grade instruments. Their data can help users respond effectively to changing indoor conditions. Further details on their performance and experimental results from real-world tests are presented in Section 5.

In the next section, through real-world experimentation, we present and compare the performance of three IoT-based commercial AQM devices currently available in the UK market. The graphical results-based critical analysis of the output data from these commercial AQM systems is the focus with strengths and weaknesses highlighted.

## 5. Performance Evaluation of AQM Systems

In this section, data collected in various indoor environments from three different commercially available AQM devices is analysed. These monitors include the ubibot aqs1 smart air quality sensor [84], temptop 1000s+ [85], and the Amazon smart AQM device [86]. Results are presented in graphical format, with colour coding to indicate different air quality levels: green colour for good air quality, yellow colour for fair air quality, orange colour for poor air quality, and red colour for terrible air quality [87]. A total of three experiments were conducted to assess indoor air quality under different conditions. The first experiment was focused on high pollution levels during cooking activities. The second experiment involved AQM in a bedroom occupied by two adults, with all windows shut to establish an environment without ventilation. The third experiment was similarly set in a bedroom with two occupants but with a window open to allow ventilation.

### 5.1. Impact of Cooking on Air Quality Using Three Commercial Sensors

**Figure 14:**
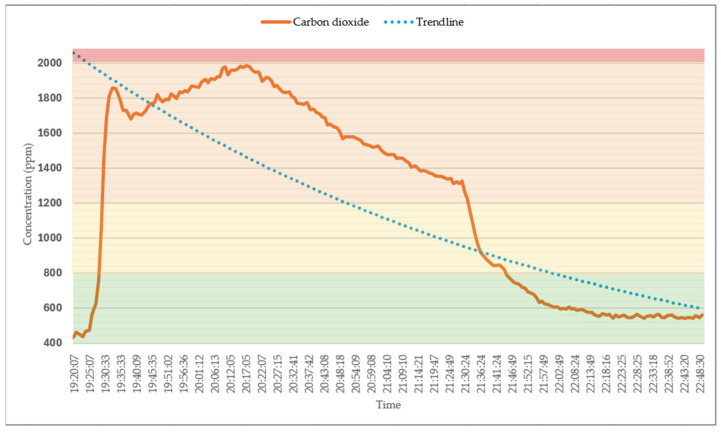
Ubibots device—CO_2_ level increases while cooking.

Figure 14 illustrates the elevated CO_2_ levels observed during cooking on a conventional gas stove in an unventilated environment. The figure depicts a sharp increase in the CO_2_ levels with the initiation of cooking. This trend is depicted by an exponential curve. CO_2_ levels continued to rise until the range hood was activated at 20:10, which led to a gradual decline in concentration beginning at 20:19. This reduction continued in a nearly linear fashion until a window for ventilation was opened at 21:28 to further ventilate the area. This action resulted in a sharp decline in CO_2_ levels, ultimately bringing it down within the optimal range for air quality.

**Figure 15:**
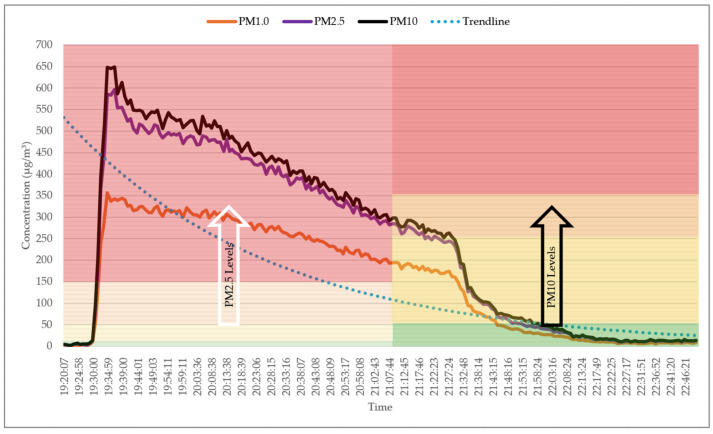
Ubibots device—PM1.0, PM2.5, and PM10 Levels During Cooking: Darker colours on the right side indicate the health hazard levels of PM10, while lighter colours on the left represent the health hazard levels of PM2.5.

The graph in Figure 15 illustrates the levels of PM1.0, PM2.5, and PM10 during cooking activities, along with a trendline indicating an exponential change in concentrations over time. Initially, when cooking activities began, the levels of PM rose sharply within a few minutes to hazardous levels. Before 20:10, when no ventilation was present, the decline in PM levels was gradual, likely due to the PM diffusing throughout the air. The activation of the range hood at 20:10 resulted in a steeper decline in PM levels. However, the most significant reduction occurred at 21:28, when a window was opened for ventilation, leading to a rapid drop to safe levels. This observation suggests that opening a window provides more effective ventilation compared to using a range hood.

**Figure 16:**
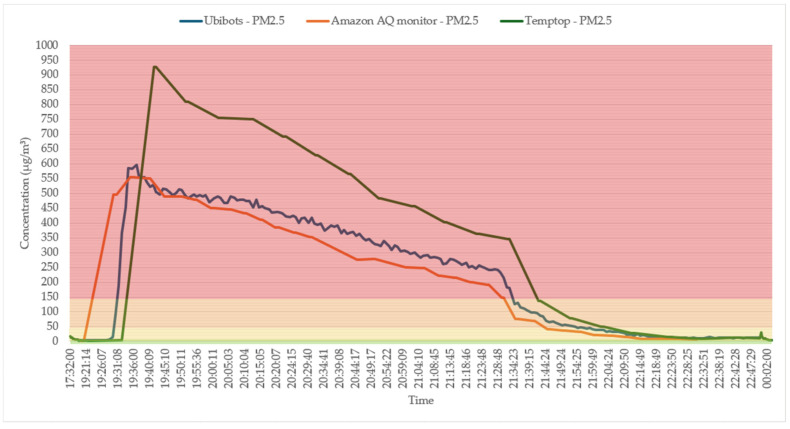
Comparison of PM2.5 Levels Measured by ubibots, temptop, and Amazon AQM devices in an identical Environment.

Figure 16 compares PM2.5 levels measured by ubibot, amazon, and temptop AQM devices. Although all AQM devices displayed similar trends, there were differences in the absolute values recorded. Temptop devices consistently showed the highest values, followed by the ubibot and then the Amazon AQM device. This discrepancy highlights the variance in sensitivity and accuracy among different AQM devices, even when placed next to each other. Notably, the Amazon device was the fastest to sense changes in the environment.

**Figure 17:**
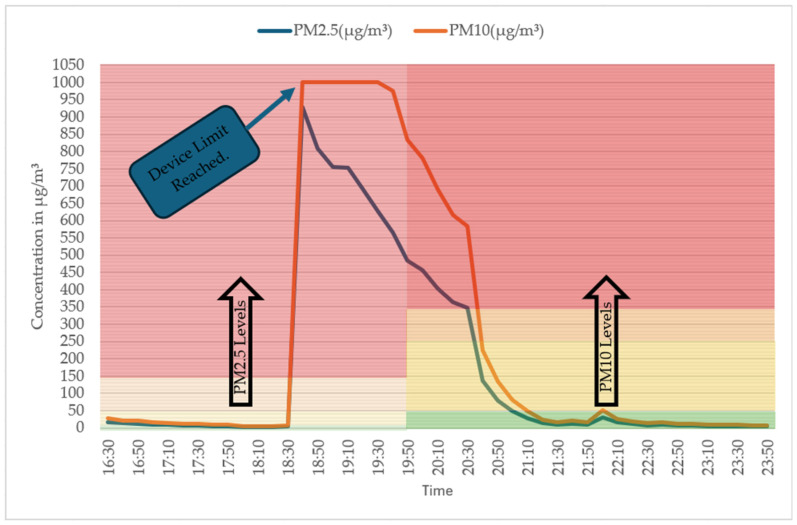
Temptop device—PM1.0, PM2.5, and PM10 levels during cooking: Dark colours on the right side indicate the health hazard levels of PM10, while lighter colours on the left side represent the health hazard levels of PM2.5.

Figure 17 illustrates the PM2.5 and PM10 levels recorded by the temtop device during the same cooking experiment. PM10 levels showed a sharp increase until the device reached its detection limit of 999 µg/m^3^. At this point, the PM10 concentration readings from the monitor plateaued because the device cannot measure beyond this limit. The user manual of the temptop 1000s+ device notes the use of a highly precise laser particle sensor to measure PM2.5 and PM10 within the range of 0–999 µg/m^3^ with a resolution of 0.1 µg/m^3^ [88].

### 5.2. Comparison Analysis of Indoor Air Quality with and Without Ventilation in a Residential Setting

**Figure 18:**
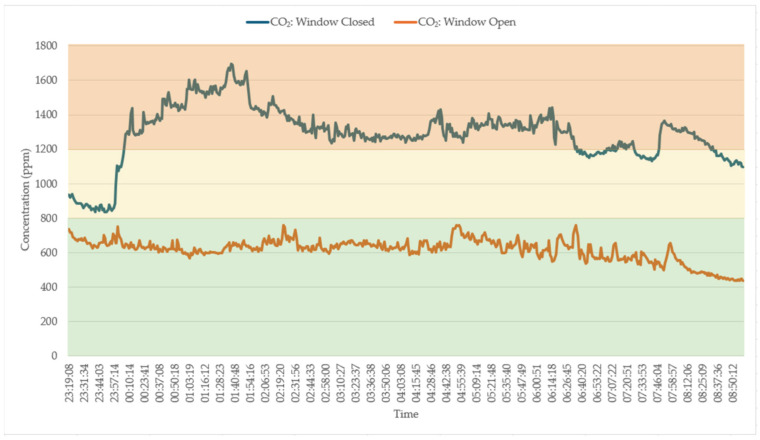
Ubibots device—Comparison of CO_2_ levels in a room with two people sleeping: Effects of open window (ventilated) vs. closed window (no ventilation).

Figure 18 depicts the CO_2_ levels recorded in a bedroom during two different sleep scenarios involving two adults. On the first occasion, all the windows were closed while the bedroom door remained open, resulting in no ventilation from the outdoor environment. This scenario led to a significant increase in CO_2_ concentrations, as illustrated in the graph. Initially, the CO_2_ levels were in the “fair” air quality range when the adults began sleeping. However, within less than 30 min, the CO_2_ levels escalated to the “poor” air quality range. The second occasion involved keeping the bedroom door open along with an open window in an adjacent room, which facilitated some degree of ventilation from the outdoors. This setup helped maintain the CO_2_ levels within the “good” air quality range throughout the night, as shown in the graph. The graph clearly indicates that when no windows were open, the CO_2_ concentrations rapidly increased, reaching “poor” levels due to insufficient ventilation. Conversely, with the window in the adjacent room open, the CO_2_ levels stayed within a healthier range, demonstrating the importance of adequate ventilation for maintaining air quality during sleep.

**Figure 19:**
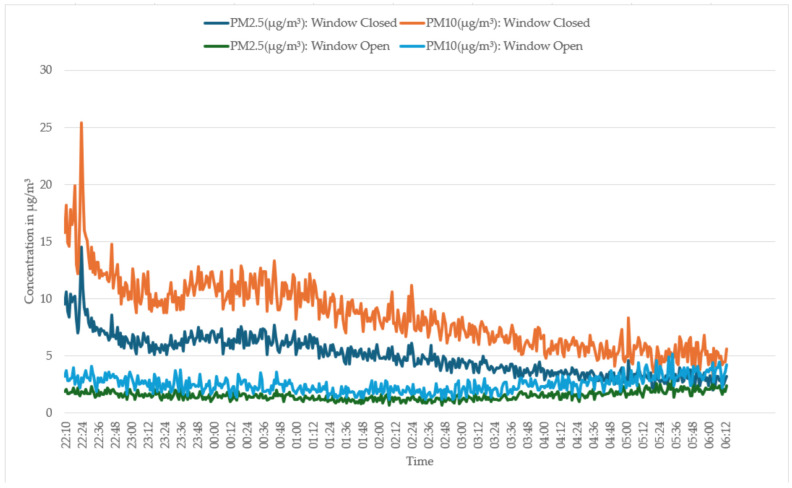
Temptop device- Comparison of PM2.5 and PM10 levels in a room with two people sleeping: Effects of open window (ventilated) vs. closed window (no ventilation).

Figure 19 presents the PM2.5 and PM10 levels recorded by the temtop device under two different ventilation scenarios in a bedroom occupied by two adults. With all the windows closed, PM2.5 levels spiked to around 14.5 µg/m^3^ and remained elevated. PM10 levels followed a similar trend, peaking near 25.4 µg/m^3^ and then remained elevated throughout the night. According to the temtop 1000s+ device’s datasheet, these values fall within the “moderate” range, indicating a decline in air quality due to limited ventilation. In the second scenario, where an adjacent room’s window was open, PM2.5 levels remained much lower, fluctuating between 2 and 5 µg/m^3^. Similarly, PM10 levels were also low, ranging from 3–6 µg/m^3^. These concentrations are within the “good” range, illustrating the effectiveness of increased ventilation in maintaining better air quality. The data emphasise that without adequate ventilation, both PM2.5 and PM10 concentrations increase, leading to diminished air quality. Conversely, improving ventilation by opening a window in an adjacent room significantly reduces PM levels, resulting in a healthier indoor environment for the occupants.

### 5.3. Sensor Calibration

Accurate calibration of AQM sensors is critical to ensure precision and reliability of measurements. Sensors, particularly low-cost ones used in IoT applications, can exhibit errors due to environmental conditions such as temperature, humidity, and cross-sensitivity to other pollutants. Sensor calibration methodologies can be broadly divided into three types including co-location calibration, laboratory calibration, and in-field calibration. Each approach has its strengths and limitations regarding accuracy and reliability compared in Table 5. yesCo-location calibration method involves placing air quality sensors next to high-precision reference instruments, such as regulatory-grade air monitoring stations, for a defined period under real-world environmental conditions in a field setting. This allows for a direct comparison of sensor outputs with reference measurements, enabling the development of calibration equations (e.g., linear regression or machine learning algorithms) [89]. In the laboratory method, the sensor is placed in a test chamber with controlled parameters, including pollutant concentration, temperature, and humidity. Known reference gases or particles (e.g., NO_2_, CO, PM2.5) are introduced, and the sensor’s response is compared to the true concentration. The in-field calibration method is used after the sensors are deployed at their final monitoring location. It ensures that the sensors are adjusted to match the specific environmental and operational conditions of the deployment site considering humidity, microclimate, or site-specific pollution sources [90].

## 6. Future Research Directions in AQM

### 6.1. STM32 Based AQM Systems

The majority of researchers have utilised Arduino microcontrollers or their derivatives in their research. It reported that 86% of the current indoor AQM systems rely on Arduino or Arduino-oriented microcontrollers [91]. While Arduino is a popular choice, particularly for learning and prototyping, it presents many restrictions e.g., limited user access to control microcontroller’s power consumption and hardware peripherals, which is necessary for commercial and sophisticated AQM. Arduino-based systems result in increased power consumption on top of individual sensor’s power consumption. This inefficiency reduces device mobility, as frequent human intervention is required to maintain functionality.

In contrast, the STM32 series microcontrollers are known for their exceptional performance, rapid processing speed, affordability, and superior power efficiency [92]. These microcontrollers offer step-by-step real-time power consumption analysis, a high level of integration, user-friendly development tools, ease of conversion to the final product, robust hardware debugging capabilities, and superior analogue-to-digital converters with higher resolution [93]. To address these challenges, future research on AQM system development should focus on better integration of advanced microcontrollers like STM32 and other emerging technologies to improve power efficiency, enhance security, and reduce the need for manual intervention, ultimately leading to more robust and autonomous systems [94].

### 6.2. Mobile and Distributed AQM

Traditional AQM systems are installed as static devices. The static installation base AQM is insufficient in providing high spatial and temporal resolution air quality data of the surrounding environment. In contrast, mobile and distributed AQM systems are increasingly attracting the attention of researchers [95,96]. However, several challenges must be overcome to develop effective mobile and distributed AQM systems, including power consumption, low-cost sensor accuracy, privacy, data processing, communication, and networking issues [97].

**Figure 20:**
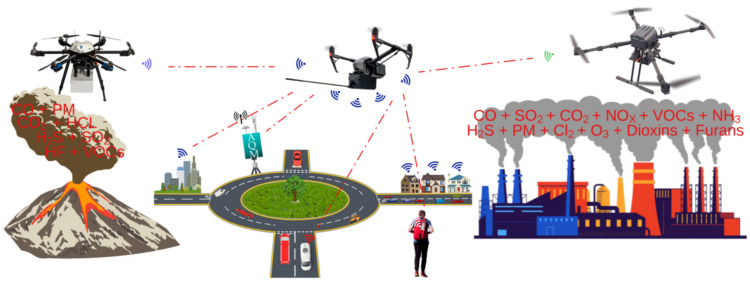
Overview of mobile and distributed AQM system.

In this direction, a deep learning algorithm using variational graph autoencoders has been presented to estimate air pollution levels based on actual sensor data and road network topology [98]. The device was mounted on a postal truck and utilised opportunistic calibration to autonomously calculate calibration parameters. Figure 20 presents a conceptual framework that integrates drones equipped with sensors for the detection of pollutants originating from both natural (e.g., volcanic emissions) and anthropogenic (e.g., industrial activities) sources. It demonstrates the use of air quality sensors mounted on various platforms, including unmanned aerial vehicles, automobiles, wearable devices, and static installations such as roadside units and indoor environments (e.g., homes and commercial buildings). While this distributed and dynamic monitoring approach offers great potential for real-time air quality tracking across diverse environments, several challenges remain unaddressed. These include ensuring the efficiency and accuracy of pollutant detection, optimising communication between the devices, and addressing infrastructure requirements for seamless and effective connectivity among these mobile and stationary nodes.

### 6.3. Optimising Sensor Calibration for AQM

Sensor drift is a significant challenge that impacts the accuracy of AQM systems [99]. Unit-to-unit variability further complicates the calibration process, making it difficult to ensure consistency. Applying the same calibration across all devices worsens data quality issues since the extent of drift varies even among sensors of the same type. Combined errors from baseline drift and sensitivity drift add to the complexity of the situation. As a result, robust optimisation algorithms for dynamically calibrating low-cost AQM systems to mitigate sensor drift are increasingly attracting researchers [100].

**Figure 21:**
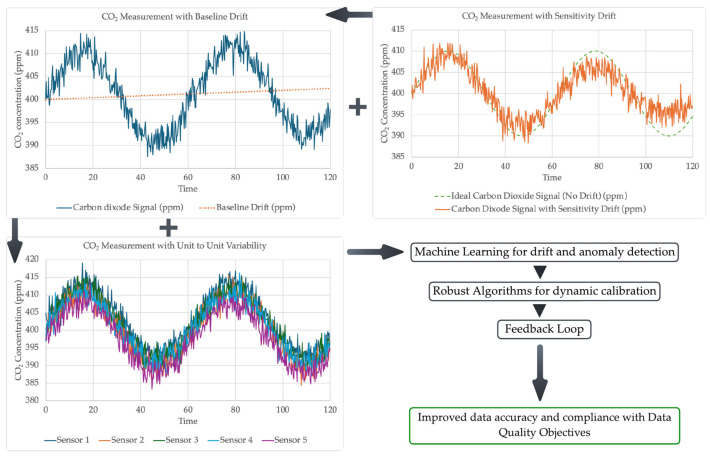
Overview of optimisation of sensor calibration for addressing measurement deviations.

In this context, an intelligent calibration method for air pollution monitoring during extreme events has been presented using a Bayesian framework [101]. It is demonstrated that while black-box calibrators are more accurate, they are prone to drift during new events, whereas white-box calibrators maintain robustness. Figure 21 provides a graphical representation of various factors affecting sensor accuracy including baseline drift, sensitivity drift, and unit-to-unit variability. Representative data of a typical CO_2_ sensor output has been used for illustration purposes. The CO_2_ measurement with sensitivity drift graph contrasts the ideal signal (in green colour) against typical real-world data as the sensor’s sensitivity changes over time. As the sensor ages, the deviation in different sensing regions becomes more prominent. The CO_2_ measurement with baseline drift graph illustrates how the sensor’s baseline measurement shifts either up or down over time, contributing to inaccuracies as the sensor ages. The CO_2_ measurement with unit-to-unit variability compares the example output of five different sensors of the same model, demonstrating how data can vary significantly between units, even under identical conditions. To mitigate these issues, modern techniques such as robust and optimal machine learning algorithms must be employed. These methods can dynamically adjust sensor calibrations, improving the data accuracy and reliability of low-cost AQM sensors.

### 6.4. Advances in Sensing Materials

The rise of novel sensing materials and fabrication techniques has created new opportunities for improving the efficiency of AQM devices. Sensing materials such as metal oxides, organic compounds, and nanomaterials are gaining significant interest from researchers [102]. Challenges such as integrating diverse nanomaterials with sensors, ensuring compatibility and stability in different environments, improving sensitivity and selectivity, and addressing the high-temperature dependence of metal-oxide sensors must be addressed to develop effective AQM systems.

In this aspect, a two-dimensional monolayer composite nanomaterial has been developed by doping hexagonal boron nitride with a nano-graphene domain [103]. It creates a highly sensitive SO_2_ gas sensor that operates based on changes in conductivity and remains effective in high moisture environments. Similarly, a metal-oxide-based gas sensor array has been presented composed of nickel oxide-Au, copper oxide-Au, and zinc oxide-Au thin films for the detection and quantification of VOCs [104]. It has also utilised machine learning to enhance accuracy in gas classification and concentration prediction.

## 7. Conclusions

The multidisciplinary synergy between advanced pollutant sensor design techniques and IoT solutions provides a robust platform for researchers in both fields to broaden their expertise. This study supports the purpose-built design and effective integration of innovative sensors with IoT infrastructures, and vice versa. Through detailed case studies, this research highlighted the advanced methodologies currently employed in both disciplines. This study effectively bridges the knowledge gap between sensor design and IoT technology, fostering mutual advancements in both fields. Its significance is underscored by the integration of concepts such as OTFT and York-cell ceria-based gas sensor fabrication with IoT frameworks. In this study, a wide array of key research papers from both fields are discussed. It compares sensor design and fabrication techniques for detecting gases such as NH_3_, CO_2_, H_2_S, Radon, and NO_2_. It also explores IoT architectures, including participatory methods, adaptive calibration, microclimate monitoring, and multi-point systems.

The experiments in this paper revealed high PM and CO_2_ levels in typical living environments, posing potential health risks. The comparative analysis of the output data from different devices demonstrated notable discrepancies, particularly in the Total VOC readings, which varied widely across all the devices tested. Future research can benefit from incorporating novel manufacturing practices for other available gas sensors. Additionally, integrating IoT technologies that employ innovative methods to address challenges like energy efficiency can further enhance research efforts. The future of AQM lies in the continued convergence of advanced materials science, IoT technology, and smart data analytics, promising a healthier and more informed world.

## Figures and Tables

**Table 1:** In-depth comparative analysis of sensor design parameters from various studies.

Study	Gas	Active Material	Active Channel Material Type	Fabrication Technique	Threshold Voltage V_TH_	Mobility (cm^2^/V⋅s) Range	Sensing Response	Testing Environment	Gate Type	Response Time	Recovery Time	Limit of Detection
[44]	NH_3_	P3HT/ MoS_2_	Nanocomposite of polymer (P3HT) and 2D material (MoS_2_).	FTM	−3.78 V (0 ppm) to −10.71 V (100 ppm)	0.1408 (0 ppm) to 0.1446 (100 ppm)	63.45% at 100 ppm	Closed chamber with an ambient environment.	highly p-doped silicon wafer.	-	-	~1 ppm
[45]	NH_3_	P3HT	P3HT polymer	Spin coating and FTM	−0.279 V (0 ppm) to −0.42886 V (5 ppm)	0.0995 (0 ppm) and 0.0572 (5 ppm)	47% at 5 ppm	Custom sensing setup with a temperature controller, humidity sensor, in-out valves, and a mixing fan.	p++ silicon substrate.	9 s	50 s	11.6 ppb
[46]	NH_3_	Au doped P3HT	Au nano particle doped P3HT nano composite	Fully Solution-Processed, Spin Coating and FTM	−0.1539 V (0 ppm) to −0.4980 V (5 ppm)	0.1069 (0 ppm) and 0.0627 (5 ppm)	55% at 5 ppm	10 L chamber with mass flow controller, B1500A semiconductor parameter analyser, temperature and humidity sensor (at 46% relative humidity).	Heavily boron-doped silicon substrate (p++ Si).	5 s	17 s	15.15 ppb
[47]	NH_3_	P3HT/ graphitic carbon nitride	Polymer/2D nanocomposite	Fully Solution-Processed, Spin Coating and FTM	−0.1052 V (0 ppm) to −0.3520 V (20 ppm)	0.1073 (0 ppm) and 0.041 (20 ppm)	69% at 20 ppm	Mass flow controller with sample gas cylinder, humidity sensor, mixing fan, probe setup, B1500A semiconductor parameter analyser, room temperature, 55% relative humidity, ambient air.	Indium tin oxide coated polyethylene terephthalate substrate (15 × 20 mm)	4 ± 0.5 s	36 ± 4 s	500 ppb
[48]	NH_3_	P3HT/ Graphene Oxide	Polymer/2D nanocomposite	FTM	−4.75833 V (0 ppm) to −6.15757 V (80 ppm)	0.0551 (0 ppm) to 0.02181 (80 ppm)	63% at 80 ppm	A 10 L chamber at 55% relative humidity with an inlet for precise gas insertion via an air-tight syringe and an outlet for exhaust flushing, connected to B1500 semiconductor parameter analyser.	highly boron-doped silicon substrate	44 s	82 s	278 ppb
[49]	H_2_S	P3HT/Graphene Quantum Dot	Polymer/2D nanocomposite	FTM	−13.71 V (0 ppm) to −16.21 V (25 ppm)	0.0711 (0 ppm) to 0.0060 (25 ppm)	91% at 25 ppm	A 10 L chamber at 55% relative humidity with an inlet for precise gas insertion via an air-tight syringe and an outlet for exhaust flushing, connected to B1500 semiconductor parameter analyser.	p++ Si substrate	10 s	225 s	606 ppb
[56]	CO_2_	CeO_2_	Yolk-shell nanospheres	Microwave-assisted solvothermal	-	-	1.8–2.9 times higher than commercial CeO_2_ nanoparticles.	Sensors installed in a continuous-flow Teflon chamber with DC power for temperature control, a gas mixing system regulated CO_2_ and relative humidity, a programmable electrometer measured resistance changes.	N/A	2.58 min at 2400 ppm	4.08 min at 2400 ppm	150–2400 ppm
[58]	Rn	SiO_2_	Semiconductor	Extended Tower Jazz High Voltage standard 0.18 μm CMOS	Threshold voltage variations estimate radon levels, with output voltage adjustable via trans-impedance amplifier biasing.	-	-	Exposure to an alpha radiation source and Radon gas in a controlled environment.	Polysilicon	-	-	Tested with concentrations of 200–800 Bq/m^3^.

**Table 2:** Specifications of Sensors used in Indoor IoT.

Sensor	Manufacturer	Parameter	Interface	Power Consumption	
PMS5003	Pantower China	PM2.5 based on Laser Scattering.	UART	Active Mode	Sleep Mode
				100 mA	200 µA
SHT30	Sensirion Switzerland	Temperature and Humidity	I^2^C	4.8 µW	
S80053	SenseAir Sweden	CO_2_ at response time of 20 s.	UART	18 mA average	

**Table 3:** Comparative Assessment and Evaluation of IoT-based AQM Studies.

Study	Year	Issue Addressed	Contributions	Techniques Used	Limitations
[67]	2021	Effective AQM and urban heat islands to improve public knowledge by bridging the gap between individual exposure and regional measurements.	Development of a participatory type monitoring system using low-cost sensors and IoT architectures with a web interface to visualise sensor data.	Use of small, mobile and modular sensor nodes to monitor NO_2_, PM1, PM2.5, and PM10.	Temperature and humidity measurements affected by sun exposure and wind direction, significant convergence time of sensors, high battery consumption limiting monitoring duration, calibration and accuracy issues.
[69]	2021	Evaluating the accuracy, reliability, and real-time monitoring of low-cost PM sensors.	Comprehensive review of the performance, improvement techniques, benefits, and limitations of PM sensors.	Comparison of 50 PM sensors.	High dependency on calibration, variability in performance under different conditions, high humidity sensitivity, limited calibration generalisability and need for ongoing recalibrations.
[75]	2019	Fine-grained AQM in urban areas	Vehicle-based system for high-resolution urban AQM, algorithms development and large-scale testing to demonstrate effectiveness.	500 mobile nodes for crowdsourcing and post-processing data via exponential smoothing and intelligent algorithms.	Potential inaccuracies in AQM estimates when assuming open windows reflect outside conditions, limited testing only in highly polluted cities.
[76]	2022	Stable ambient air monitoring in varying network conditions.	Introduction of a low-cost AQM system with adaptive performance stability for data transmission.	A system with metal-oxide sensors, a GPRS module, a microcontroller, and an algorithm for reducing packet loss is used to effectively monitor SO_2_, CO, NO_2_, PM, and weather conditions.	Simple adaptive algorithms may increase latency, reliant on GSM/GPRS, high power consumption, and limited field validation in varied environments.
[78]	2021	Need for real-time, multi-point indoor AQM monitoring in residential buildings.	Implementation of a multi-point IoT-based indoor AQM system and analysis of the impact of human behaviour and environmental changes on indoor air quality.	STM32 and Zigbee used for data collection and transmission, real-time data access and analysis, PM2.5 and CO_2_ monitoring.	Significant signal loss (>8%) with Zigbee over concrete walls, monitoring period was limited to one month in winter, study was confined to a single residential building.
[79]	2022	Indoor AQM and early detection of mould growth in residential buildings.	Case study on indoor AQM and mould in an Australian suburban home. Found links between poor AQ, high fungal spores, and health risks. Emphasises early detection and better building regulations.	Site inspection, air testing, surface sampling for mould, 2-month indoor AQM monitoring campaign, analysis of fungal spore concentrations, and environmental parameter measurements.	Limited to one case study, short monitoring period, reliance on basic sampling instruments, and conclusions based on observational data without extensive controls.
[80]	2021	Monitoring individual air pollution exposure using portable low-cost sensors.	Developed a citizen-based air pollution monitoring system, classified data into indoor/outdoor, validated sensor data accuracy, and provided fine-grained air pollution insights.	Data classification, consistency and accuracy validation, pollution measurement campaign over wide geographic areas, 40 portable low-cost sensors to monitor CO, NO_2_, O_3_, and PM over 6 km^2^.	Low-cost sensor data accuracy, limited geographical focus, data variability due to user handling, and simple indoor/outdoor classification limit broader applicability and precision.

**Table 4:** Features comparison of three commercial IoT-based AQ monitoring devices.

Features	Amazon AQ Monitor	Ubibots AQS1 Smart AQ Monitor	Temptop 1000S+ AQ Monitor
Temperature	✓	✓	✓
Humidity	✓	✓	✓
Atmospheric Pressure	✗	✓	✗
PM1.0	✗	✓	✗
PM2.5	✓	✓	✓
PM10	✗	✓	✓
VOC	✓	✓	✓
Formaldehyde	✗	✓	✓
CO_2_	✗	✓	✗
Equivalent CO_2_	✗	✓	✗
CO	✓	✗	✗
Wi-Fi	✓	✓	✗

**Table 5:** Advantages and limitations of calibration methodologies.

Calibration Methodologies	Co-Location	Laboratory	In-Field
Advantages	Provides accurate calibration under real-world conditions.	Provides highly precise calibration in a controlled environment.	Ensures high accuracy in the actual operating environment.
	Accounts for the impact of temperature, humidity, and pollutants that may not be captured in controlled environments.	Useful for identifying sensor response to specific pollutants and eliminating cross-sensitivities.	Accounts for site-specific conditions not captured in laboratory or co-location calibration.
Limitations	Accuracy can degrade when the sensor is moved to a different environment.	Calibration may not fully represent real-world conditions where external factors (e.g., temperature fluctuations, pollutant mixtures) influence sensor accuracy.	Calibration may need to be repeated periodically to address environmental changes.
	Requires prolonged exposure to ensure calibration robustness.		
	Dependent on the availability of high-precision reference stations.	Limited adaptability to dynamic field environments.	Requires access to reference instruments at the deployment site.

## Data Availability

Data are contained within the article. The data sheet will be made available on request to the corresponding author.
